# Supplementary material for: A Novel MDM2-Binding Chalcone Induces Apoptosis of Oral Squamous Cell Carcinoma
Source: Biomedicines. 2023 Jun 14;11(6):1711. doi: 10.3390/biomedicines11061711 (PMC10295897; doi:10.3390/biomedicines11061711)

A Novel MDM2 Binding Chalcone Induces Apoptosis of Oral Squamous Cell Carcinoma

Guilherme Freimann Wermelinger[1,†], Lucas Rubini[1,†], Anna Carolina Carvalho da Fonseca[2], Gabriel Ouverney[3], Rafael P. R. F. de Oliveira[4], Acácio S. de Souza[4], Luana S. M. Forezi[4], Gabriel Limaverde Sousa[5], Sergio Pinheiro[4],* and Bruno K. Robbs[1],*

[1] Basic Science Department, Health Institute of Nova Friburgo, Fluminense Federal University, Nova Friburgo, Brazil.

[2] Postgraduate Program in Dentistry, Health Institute of Nova Friburgo, Fluminense Federal University, Nova Friburgo, Brazil.

[3] Postgraduate Program in Applied Science for Health Products, Faculty of Pharmacy, Fluminense Federal University, Niteroi, Brazil.

[4] Department of Organic Chemistry, Chemistry Institute, Fluminense Federal University, Niteroi, Brazil.

[5] Experimental Schistosomiasis Laboratory, Osvaldo Cruz Institute, Oswaldo Cruz Foundation, FIOCRUZ, Rio de Janeiro, RJ, Brazil.

[†] These authors contributed equally to this work.

*Correspondence:

Biological Assays: Bruno K. Robbs, Fluminense Federal University, Department of Basic Sciences, Nova Friburgo Health Institute, CEP 28625-650, Nova Friburgo-RJ, Brazil. Email: brunokr@id.uff.br.

Chemistry: Sergio Pinheiro, Chemistry Institute, Federal Fluminense University, Outeiro de S. João Batista s/n, Centro, 24020-141 Niterói, RJ, Brazil; Email: spinuff@gmail.com

Keywords: Chalcone, 1,2,3-Triazole; MDM2; Oral Squamous Cell Carcinoma; apoptosis; cell cycle arrest

Supplementary Table S1: Compound shows low toxicity *in vivo*. Occurrence of morbidity, mortality, gross organs necropsy and histology results of each treated group.

Treatment	Dose mg/kg[a]	Change in body weight	Morbidity[b]	Mortality	Gross Necropsy[c]
Control	0<br>N=3	Normal	Normal	Absent	Normal
	25<br>N=3	Absent	Absent	Absent	No alteration
1f	50<br>N=3	Absent	Absent	Absent	No alteration
	100<br>N=3	Absent	Absent	Absent	No alteration

[a] Dose level for each subsequent group was determined based on the result after the previous lower dosing, [b] Morbidity symptoms were analyzed every day, two times a day and were considered as: Tremors; Convulsion; Salivation; Diarrhea; Lethargy; Coma; Signs of Pain; Mobility defect, [c] Gross organ necropsy of liver, thymus, right kidney, right testicle, heart, major lymph nodes and lung.

Supplementary Table S2:
Interaction mapping using Discovery Studio. The most favorable conformation was determined by the lowest binding energy obtained on Autodock. The Mdm2 pocketis mainly hydrophobic, which can be seen by the interaction pattern present on Nutlin-3A.

Ligant	Hydrogen bond	C-H bond	Alkyl	π-Alkyl	π-Sigma	π-π stacked
**1a (-7.94 kcal/mol)**	GLN24, TYR100	HIS96	-	LYS51, LEU54, VAL93, ILE99	LEU54, ILE99	HIS96
**1b (-8.37 kcal/mol)**	GLN24, TYR100	-	-	LYS51, LEU54, VAL93, ILE99	LEU54, ILE99	HIS96
**1c (-8.28 kcal/mol)**	GLN24, TYR100	-	ILE61, VAL75, VAL93	LYS51, LEU54, VAL93, ILE99	LEU54, ILE99	HIS96
**1f (-9.18 kcal/mol)**	GLN24, TYR100, TYR100	-	LEU54, VAL93, ILE99	LYS51, LEU54, PHE91, ILE99, ILE99	LEU54, LEU54	-
**Nutlin-3A (-9.52 kcal/mol)**	-	-	LEU54, LEU57, ILE61, VAL75, VAL93, ILE99, ILE99	LEU54, ILE61, OHE91, VAL93, HIS96, ILE99, TYR100	VAL93,	-

Supplementary Table S3: Physicochemical descriptors of new chalcone compounds, nutlin-3a and the reference chemotherapy drugs doxorubicin and carboplatin.

SwissADME	TPSA	MW (< 500)	H-bond aceptor (<10)	H-bond donnor (<5)	MLogP (<5)	Lipinski # of Violations
1a	73.8	292.34	3	1	1.29	0
1b	73.8	290.32	3	1	2.03	0
1c	83.03	322.36	4	1	1.79	0
1f	73.8	318.37	3	1	2.5	0
Nutlin-3a	83.14	583.51	5	2	3.94	1
Doxorubicin	206.07	543.52	12	6	-2.1	3
Carboplatin	126.64	371.25	6	4	-1.79	0

[a] Number of violations to the Lipinski "rule of 5": cLogP ≤ 5; MW, molecular weight ≤ 500; nON, number of hydrogen bond acceptors ≤ 10; and nOH/NH, number of hydrogen bond donors ≤ 5.

Supplementary Table S4: Predicted pharmacokinetic properties of chalcones, nutlin-3a and the chemotherapeutic agents, carboplatin and doxorubicin, using the admetSAR 2.0 server.

ADMET	Oral Bioavailability	P-glycoprotein inhibitor	P-glycoprotein substrate
1a	+ 0.76	- 0.77	- 0.85
1b	+ 0.71	- 0.75	- 0.88
1c	+ 0.67	- 0.66	- 0.63
1f	+ 0.67	- 0.54	- 0.72
Nutlin-3a	+ 0.53	+ 0.90	+ 0.82
Doxorubicin	- 0.91	- 0.92	+ 0.95
Carboplatin	- 0.60	- 0.99	- 0.99

Compounds spectra

Figure S1. IR (KBr) of ethyl 4-(4-(hydroxymethyl)-1*H*-1.2.3-triazol-1-yl)benzoate

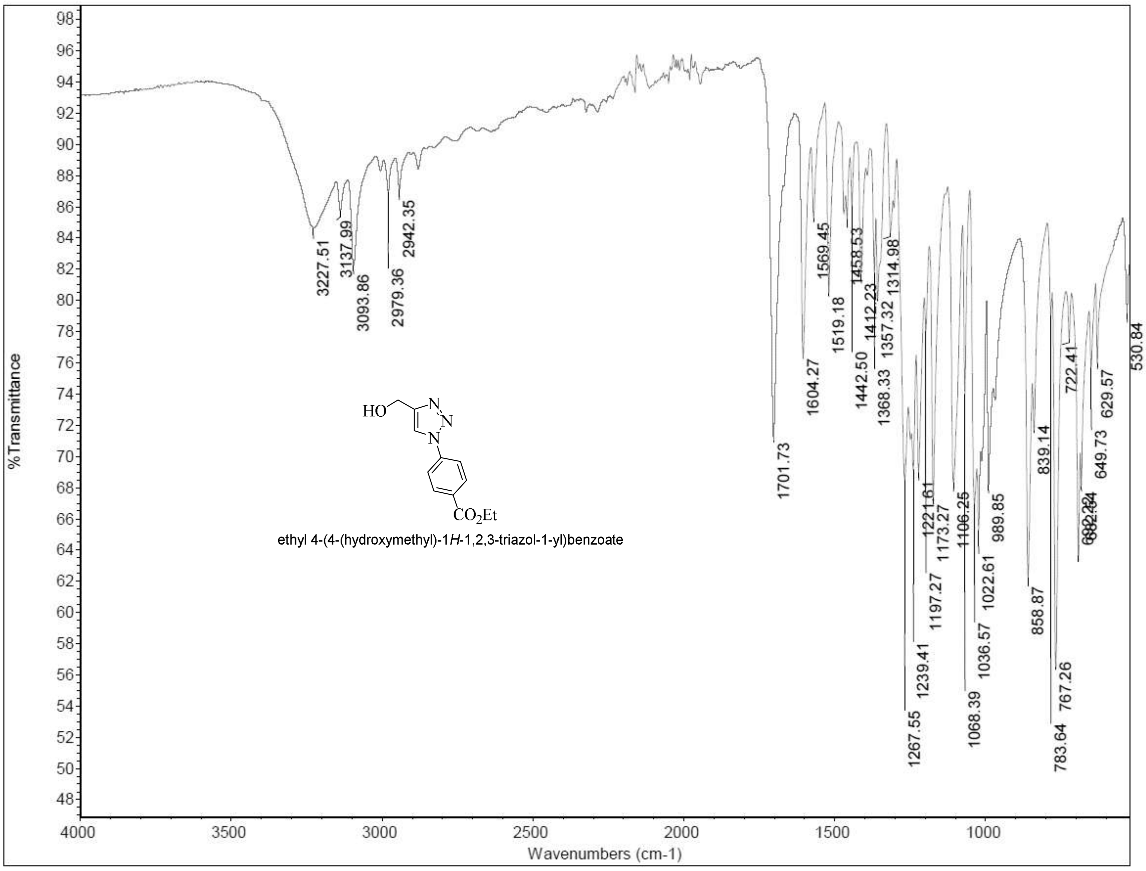

Figure S2. ^{1}H NMR (500 MHz. $CDCl_3$) of ethyl 4-(4-(hydroxymethyl)-1*H*-1.2.3-triazol-1-yl)benzoate

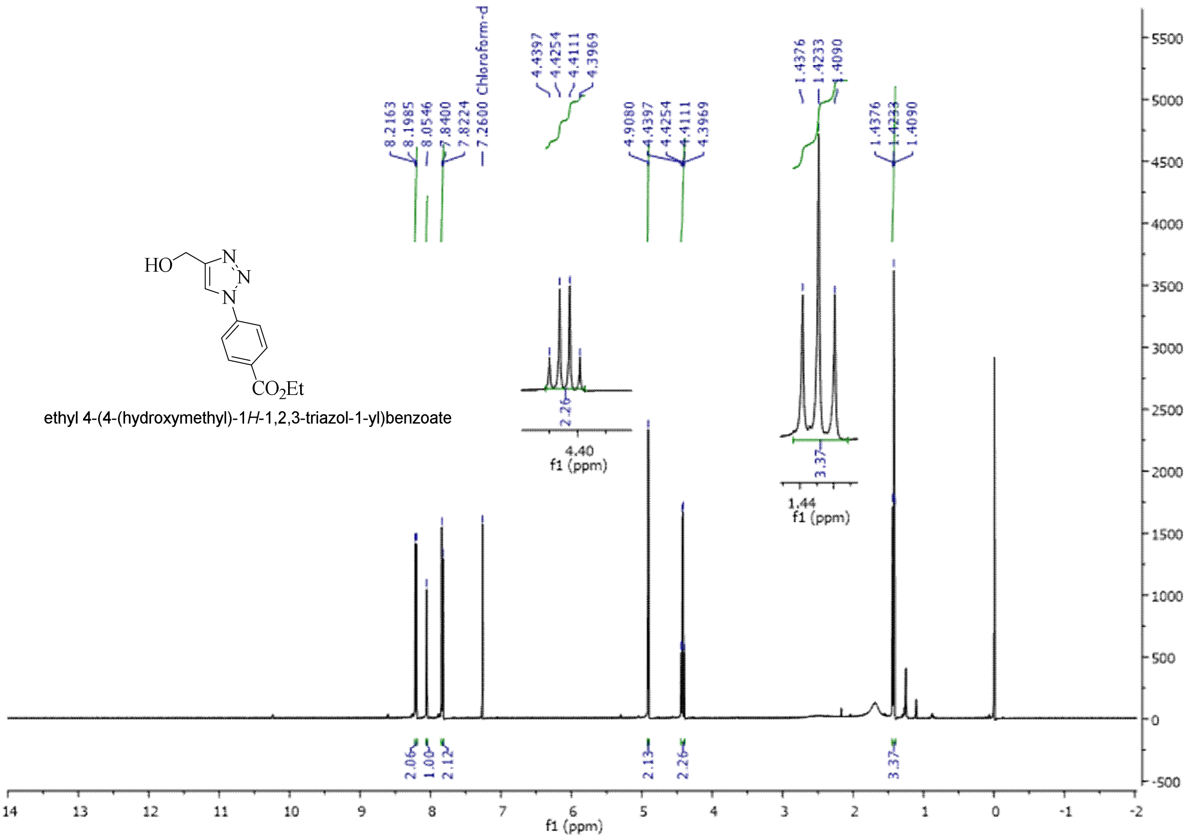

Figure S3. Expanded ^{1}H NMR (500 MHz. $CDCl_3$) of ethyl 4-(4-(hydroxymethyl)-1*H*-1.2.3-triazol-1-yl)benzoate

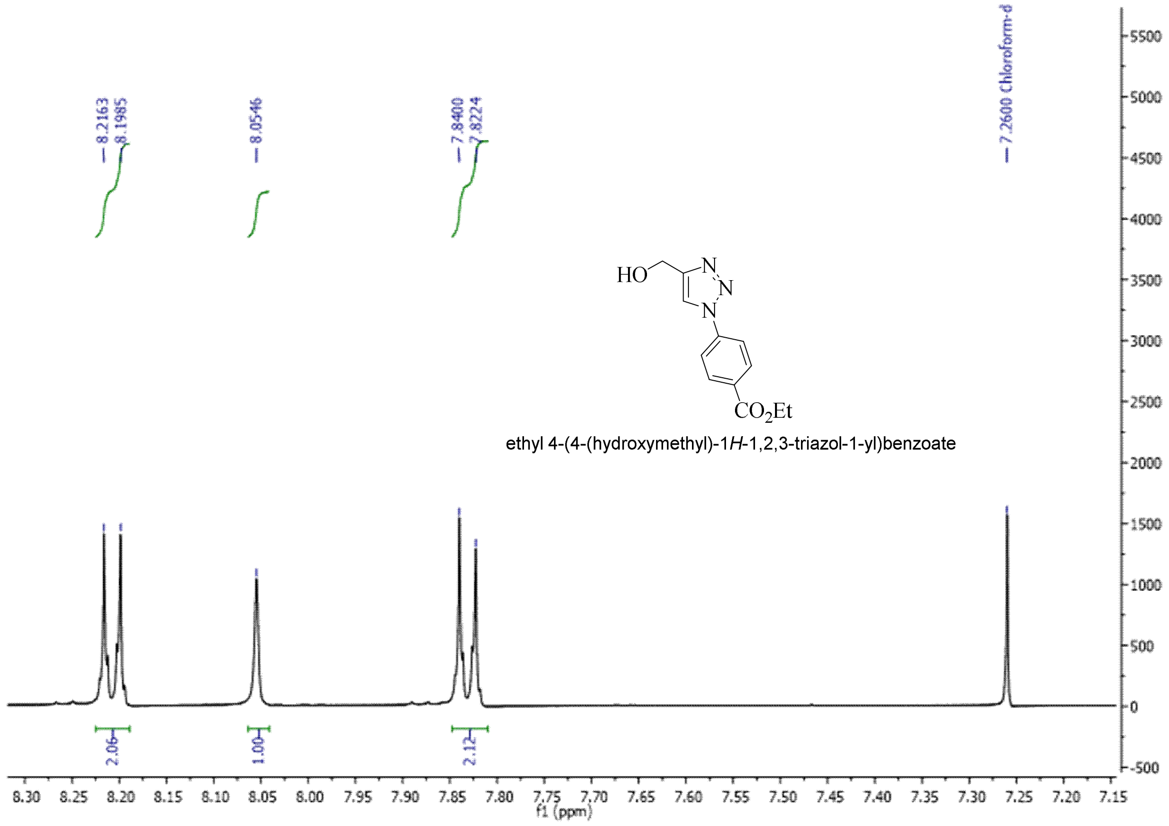

Figure S4. IR (neat) of (1-(2.6-dimethylphenyl)-1*H*-1.2.3-triazol-4-yl)methanol

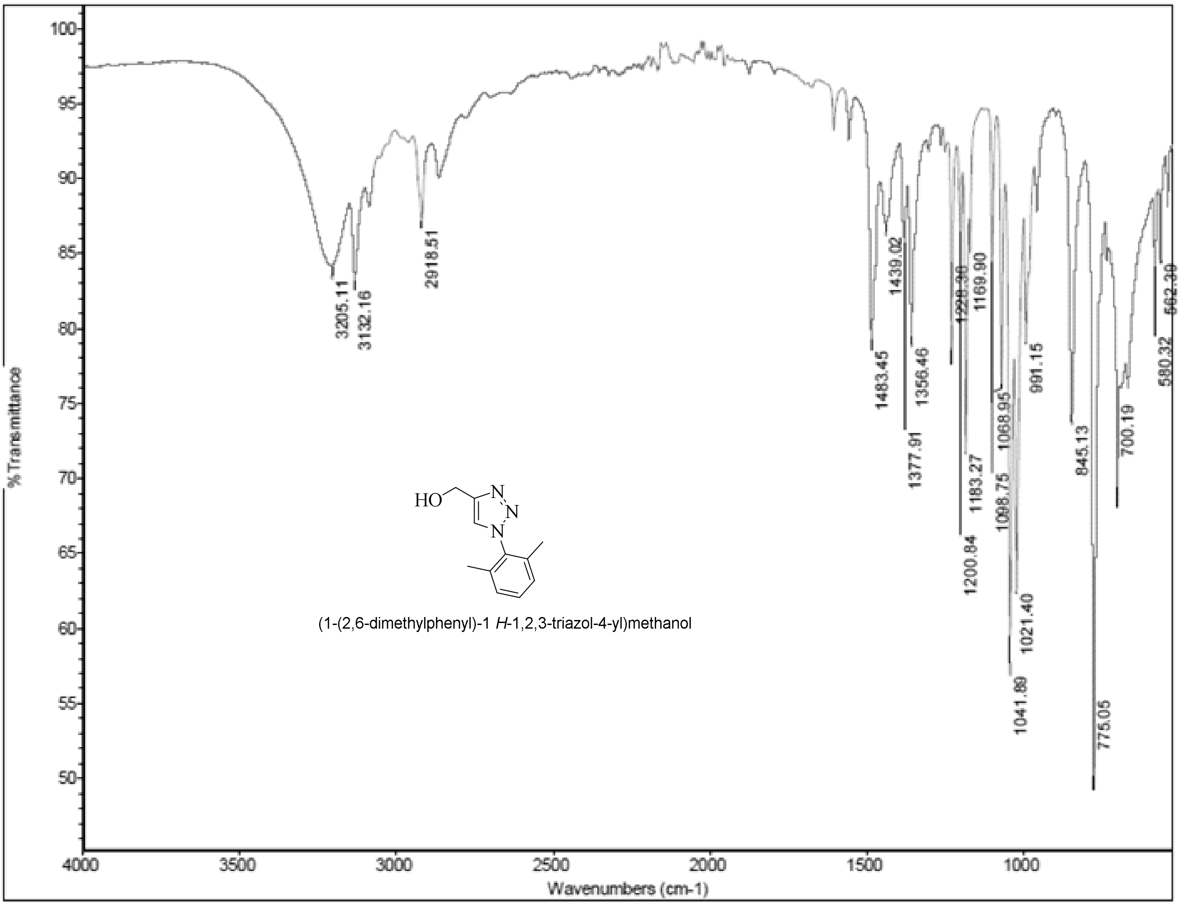

Figure S5. ^{1}H NMR (500 MHz. acetone-d6) of (1-(2.6-dimethylphenyl)-1*H*-1.2.3-triazol-4-yl)methanol

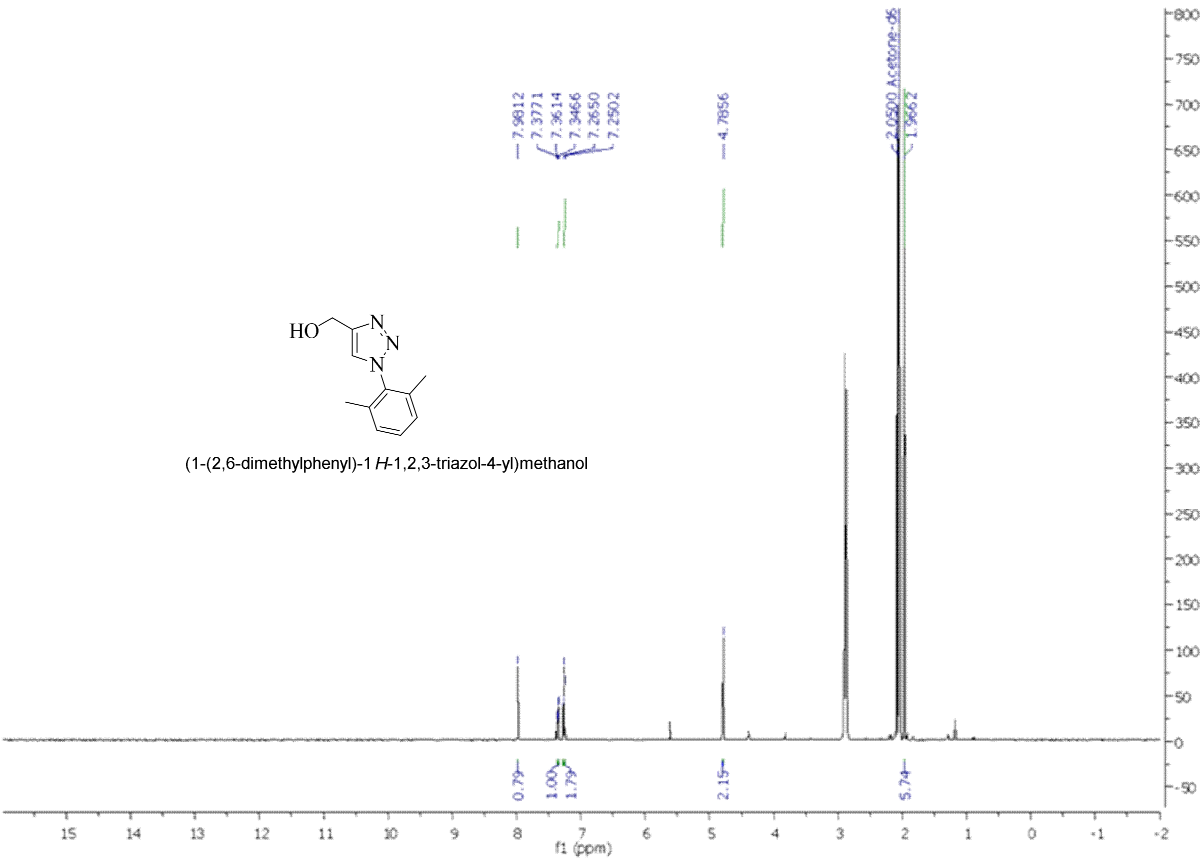

Figure S6. Expanded ^{1}H NMR (500 MHz. acetone-d6) of (1-(2.6-dimethylphenyl)-1*H*-1.2.3-triazol-4-yl)methanol

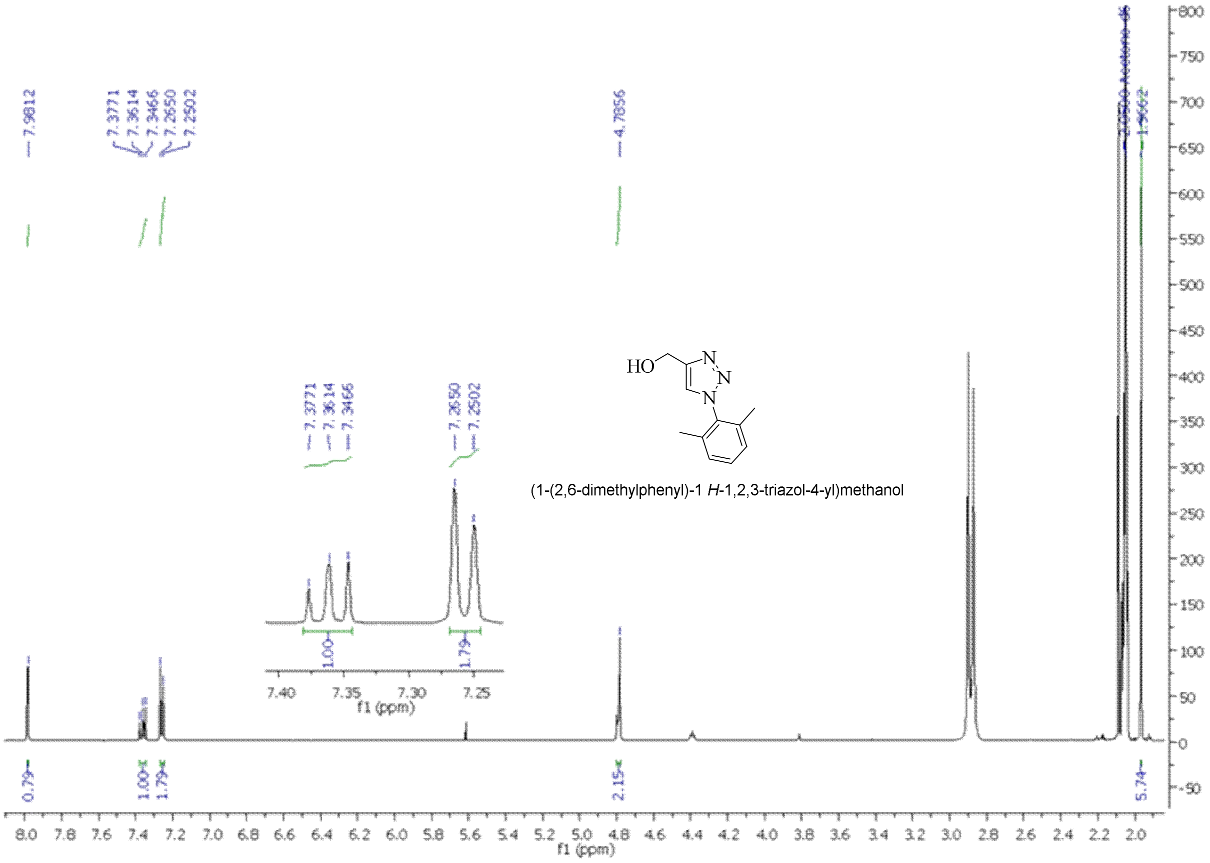

Figure S7. IR (KBr) of compound 2e

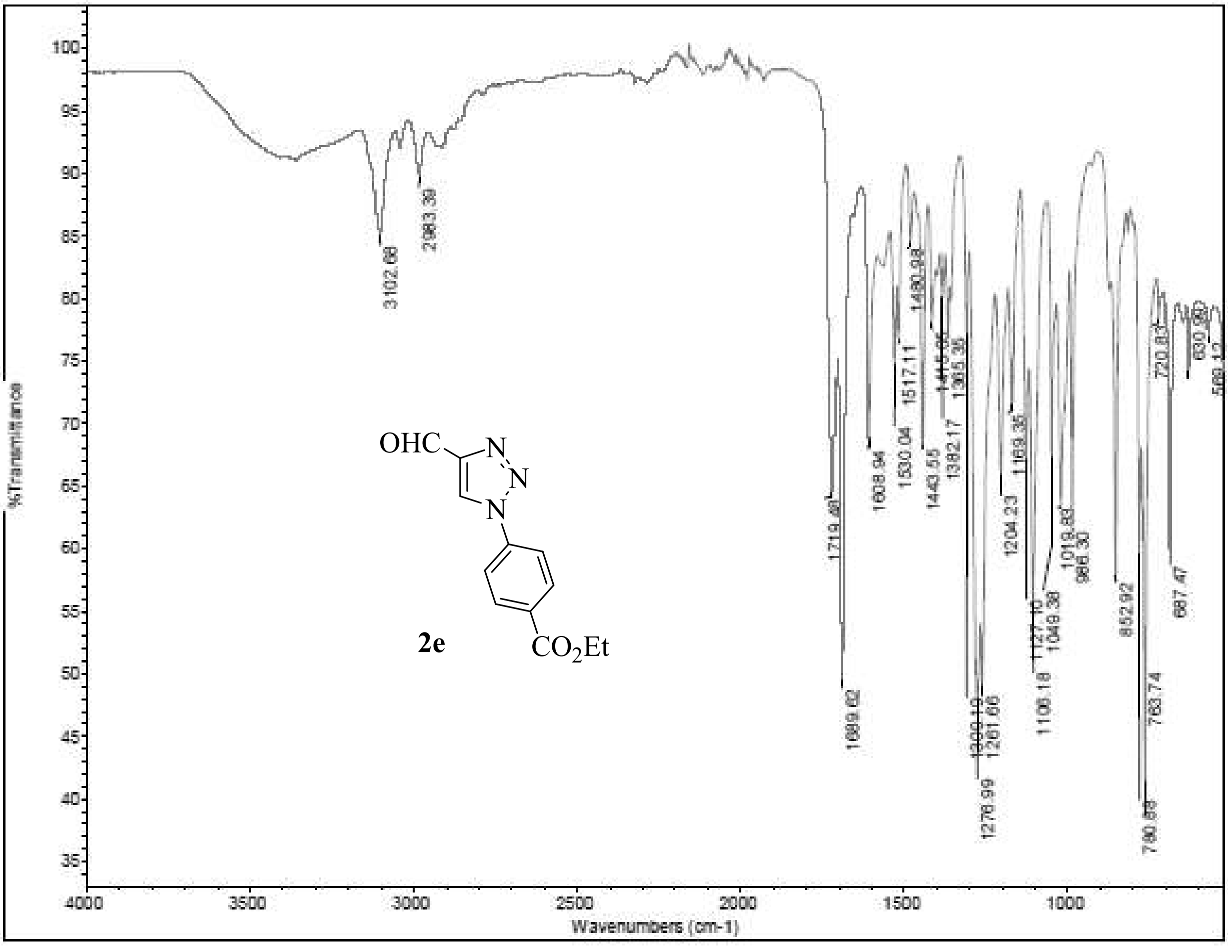

Figure S8. ^{1}H **NMR (500 MHz. $CDCl_3$) of compound 2e**

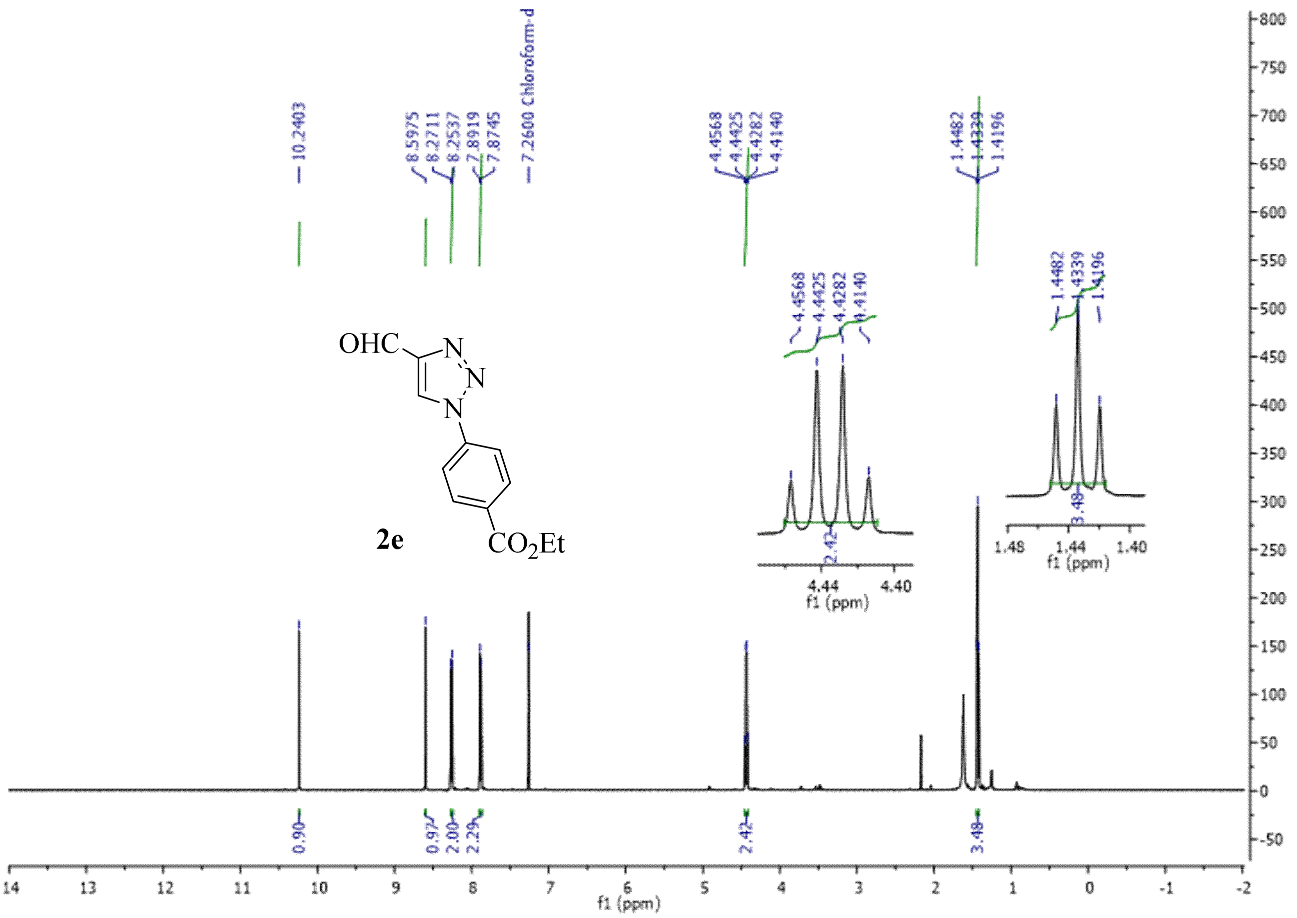

Figure S9. Expanded ^{1}H NMR (500 MHz. $CDCl_3$) of compound 2e

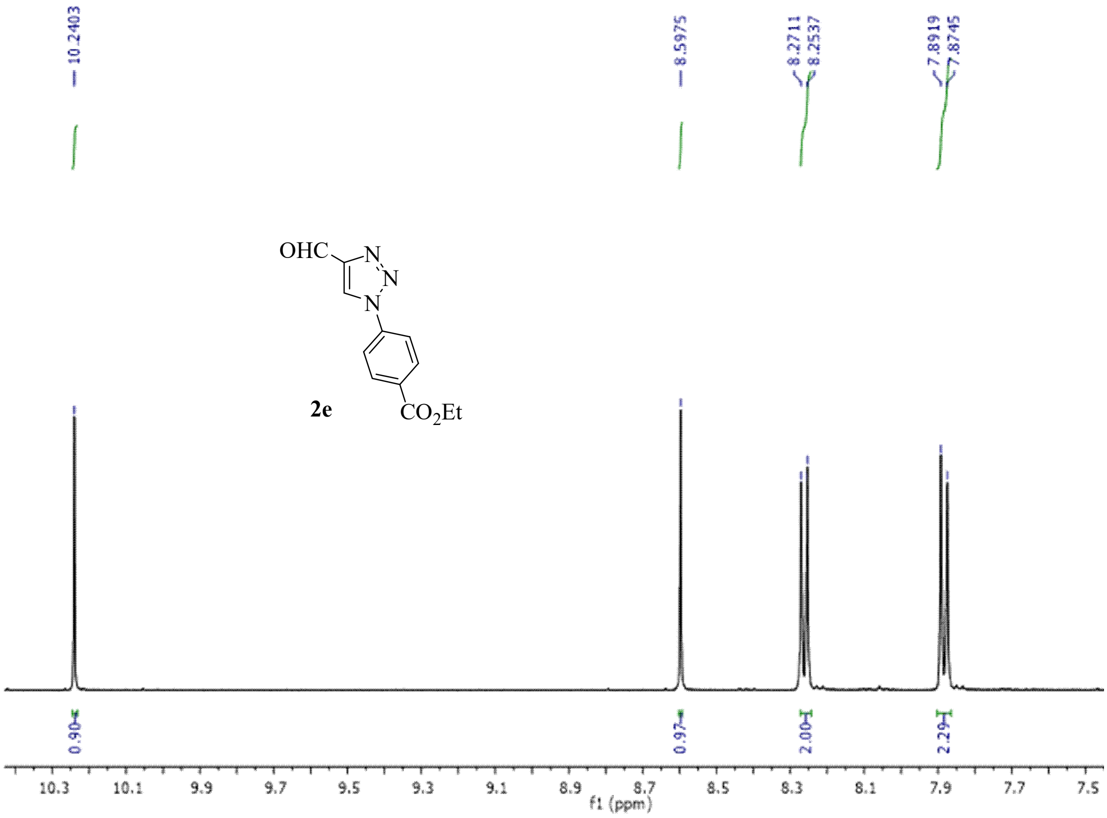

Figure S10. IR (KBr) of compound 2f

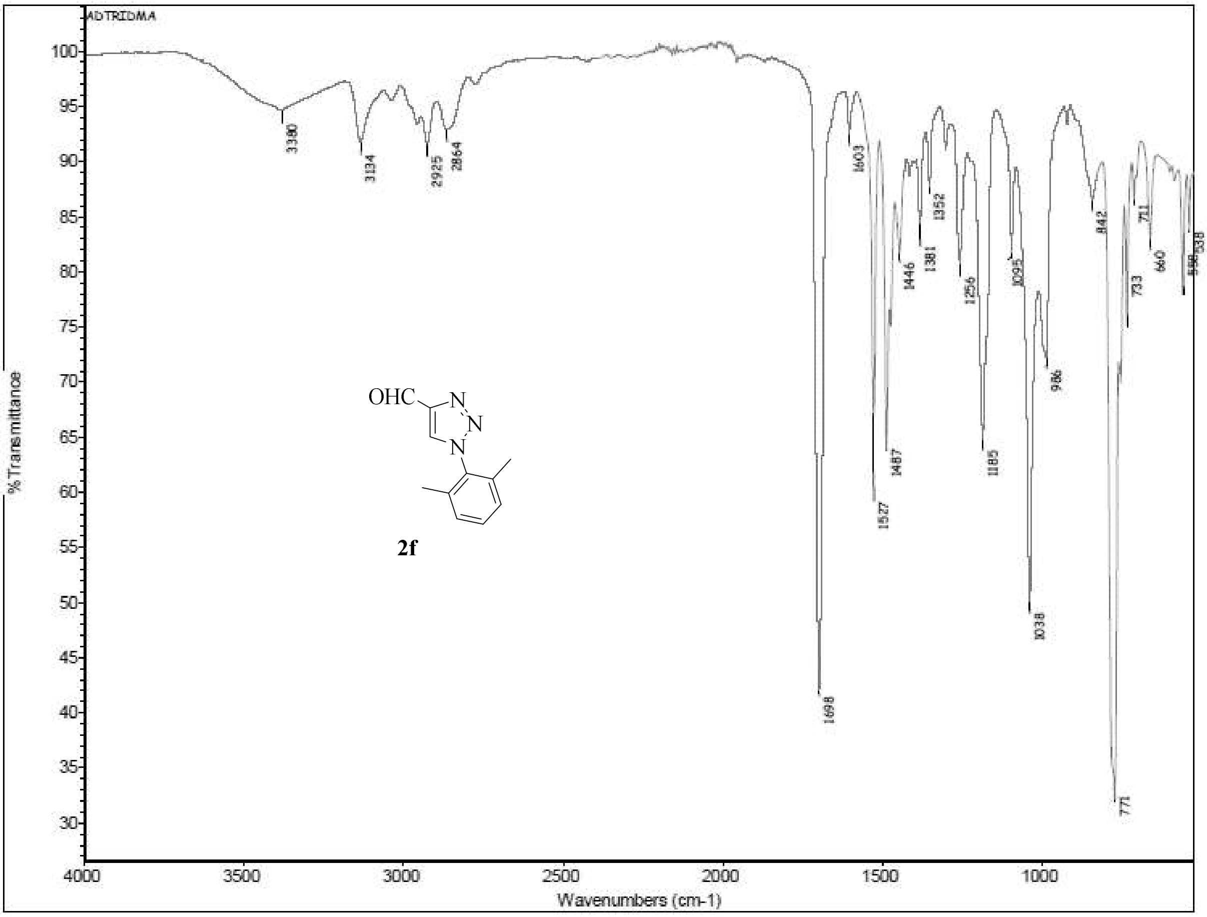

Figure S11. ^{1}H NMR (500 MHz. acetone-d6) of compound 2f

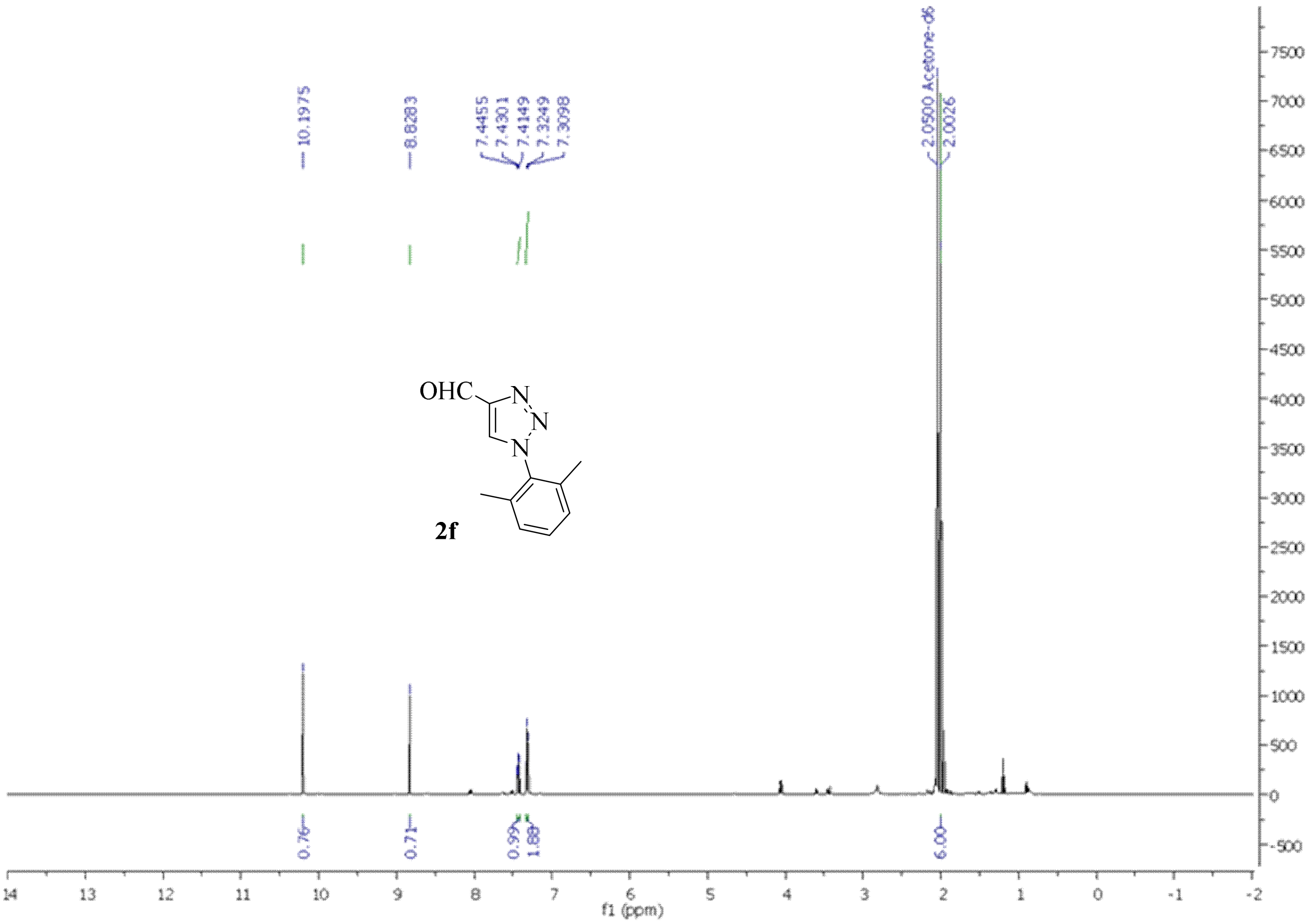

Figure S12. Expanded ^{1}H NMR (500 MHz. acetone-d6) of compound 2f

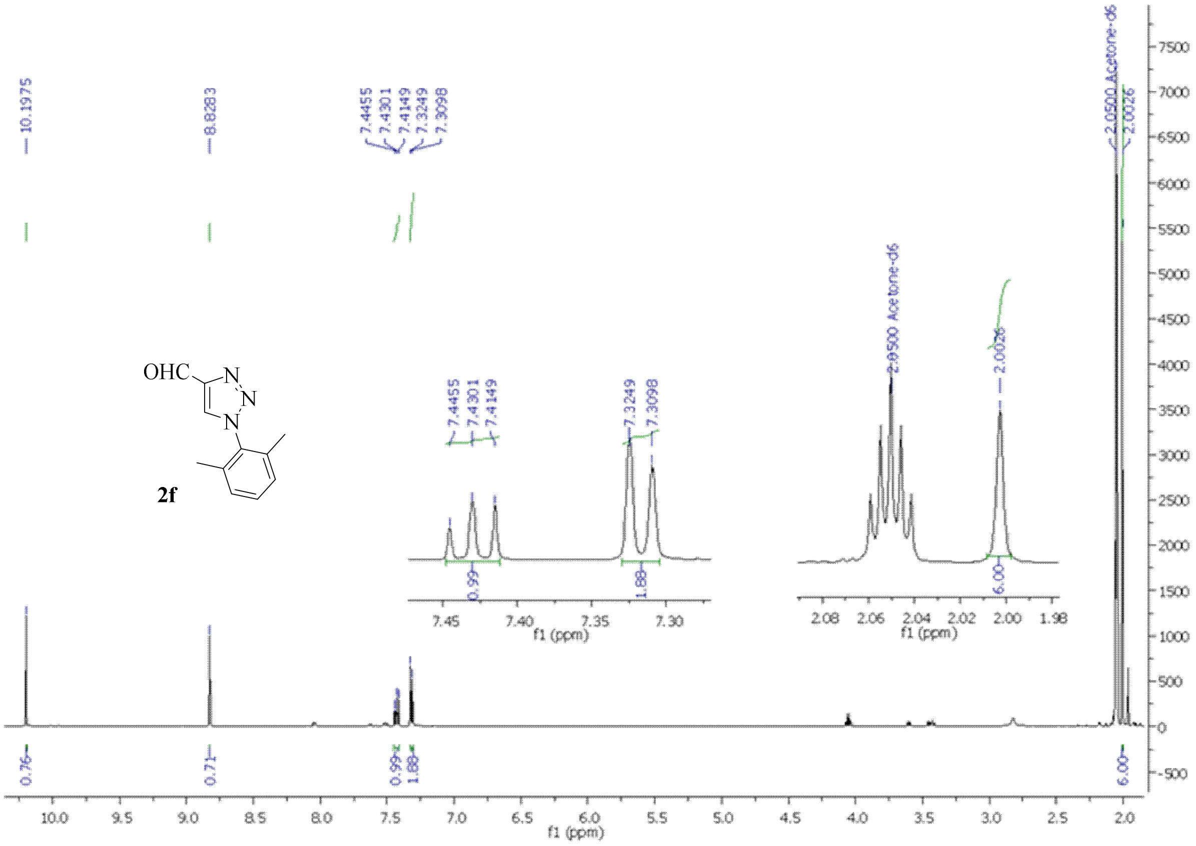

Figure S13. IR (KBr) of compound 1a

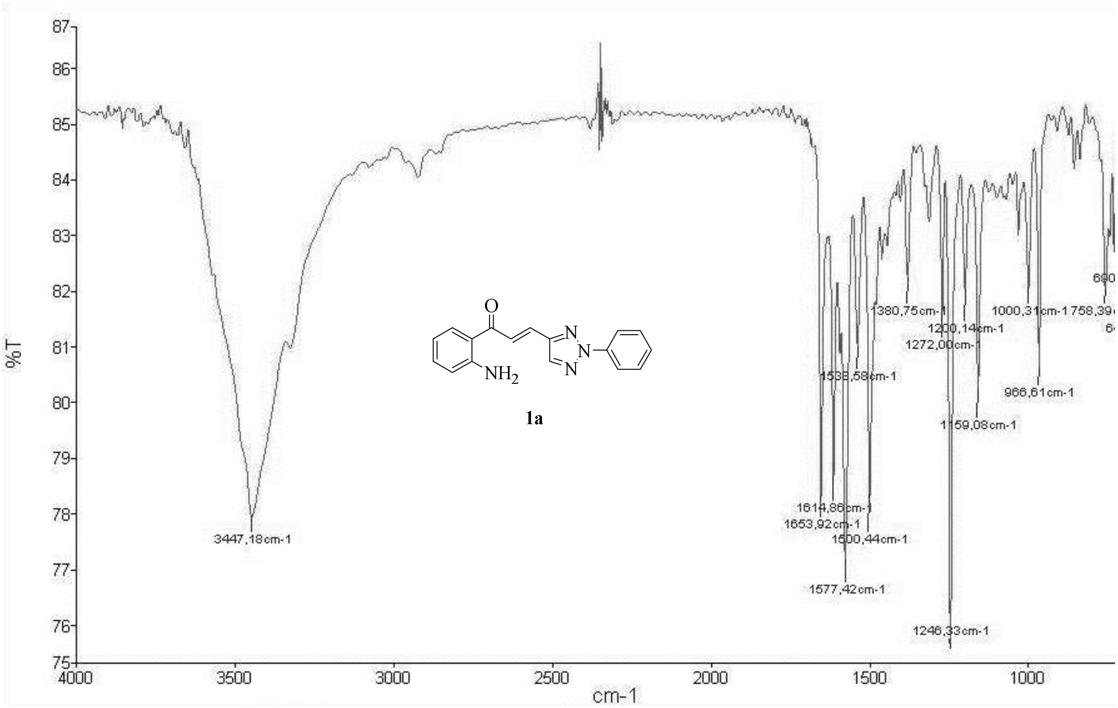

Figure S14. ^{1}H NMR (500 MHz. DMSO-d6) of compound 1a

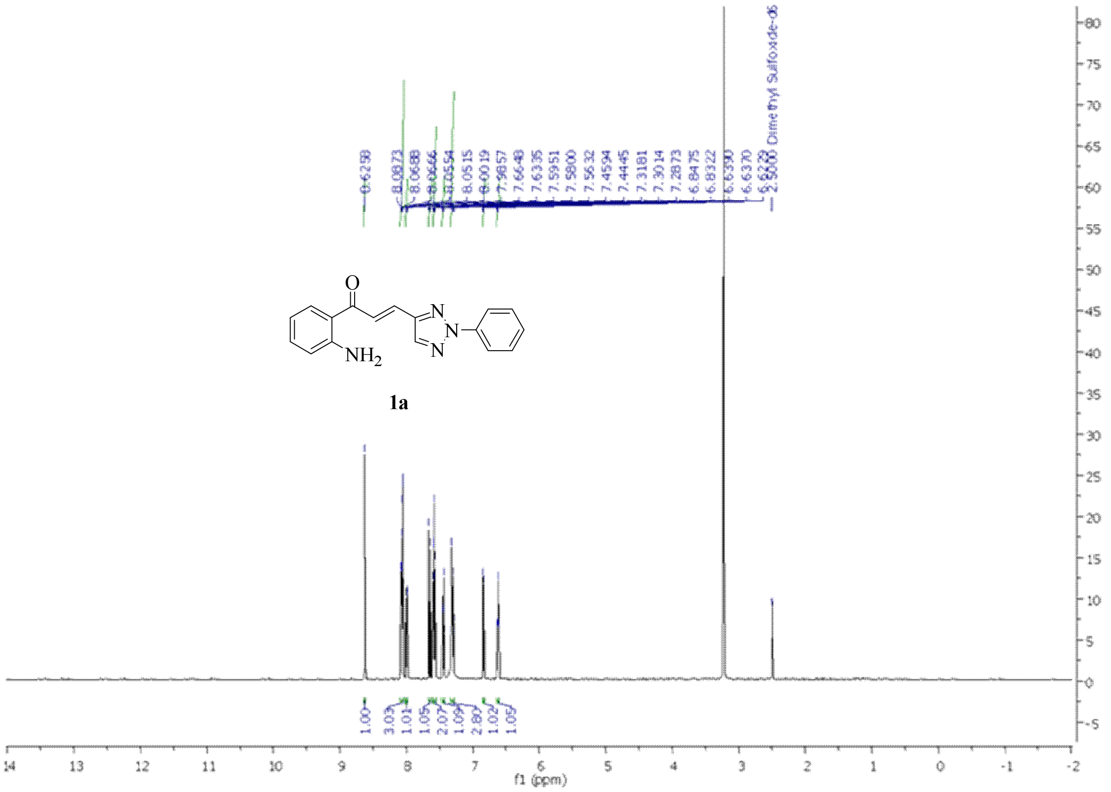

Figure S15. Expanded ^{1}H NMR (500 MHz. DMSO-d6) of compound 1a

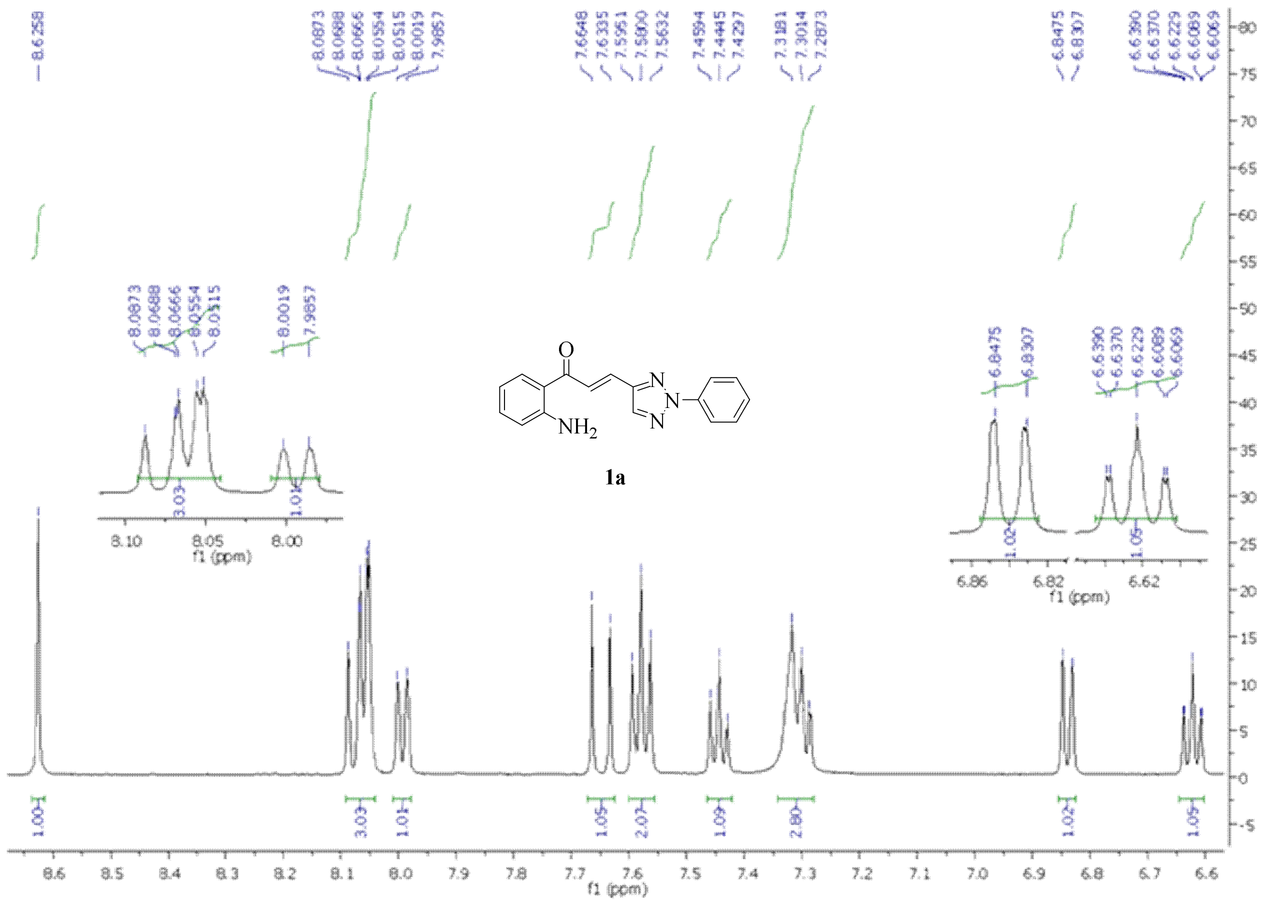

Figure S16. ^{13}C NMR/APT (125 MHz. DMSO-d6) of compound 1a

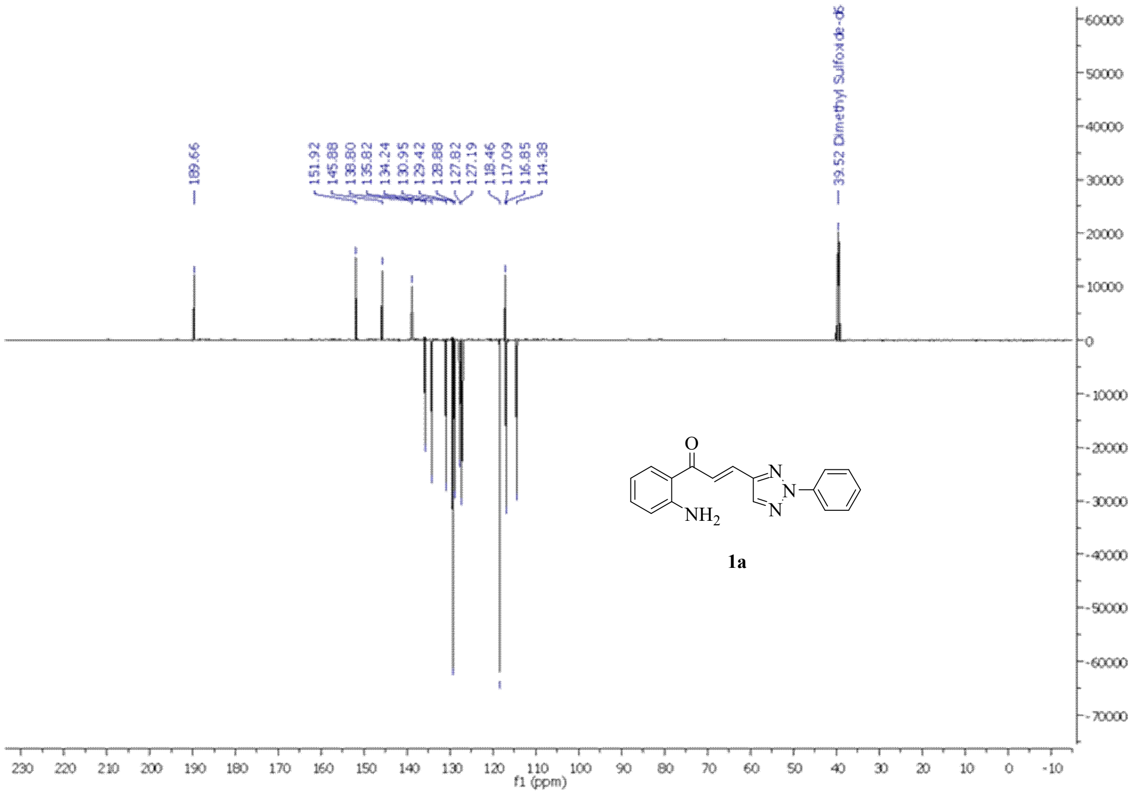

1a

Figure S17. Expanded ^{13}C NMR/APT (125 MHz. DMSO-d6) of compound 1a

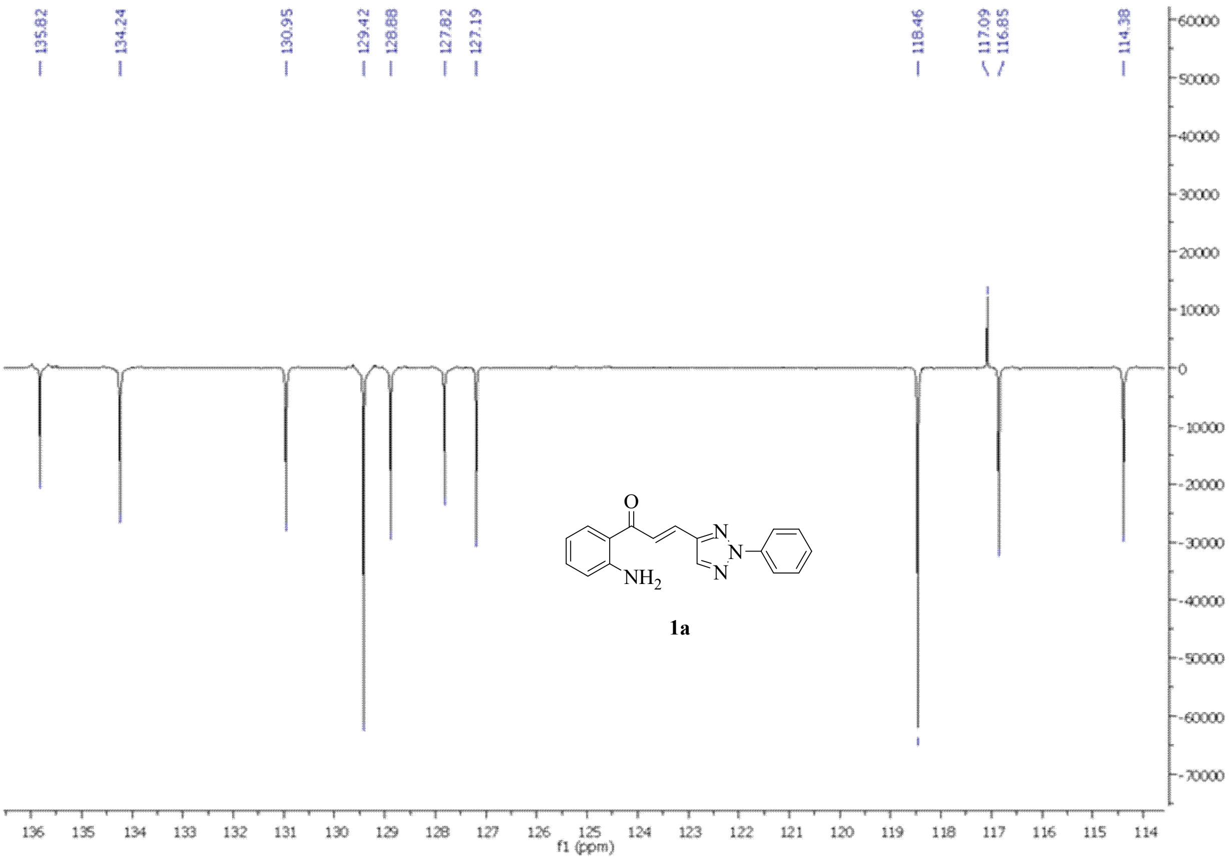

Figure S18. HSQC (DMSO-d6) of compound 1a

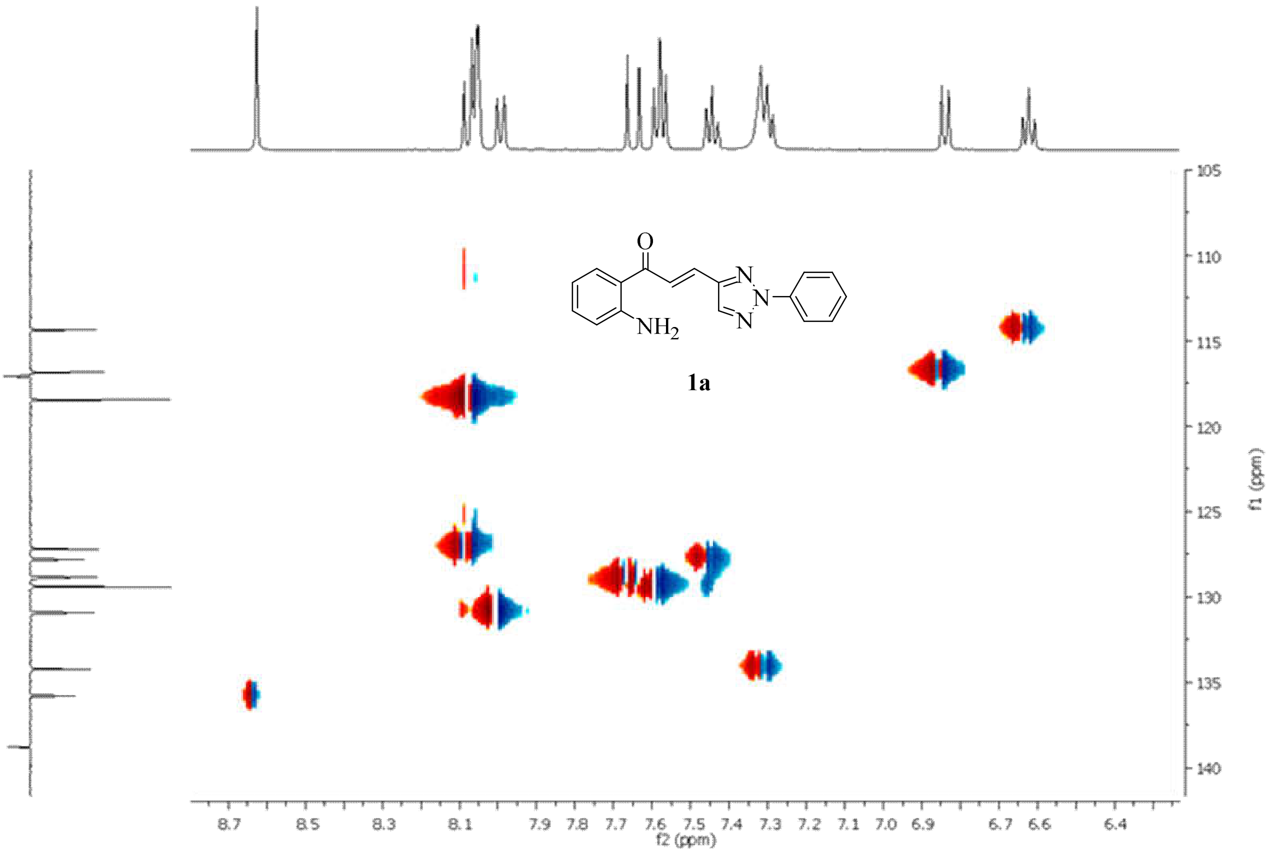

Figure S19. Expanded HSQC (DMSO-d6) of compound 1a

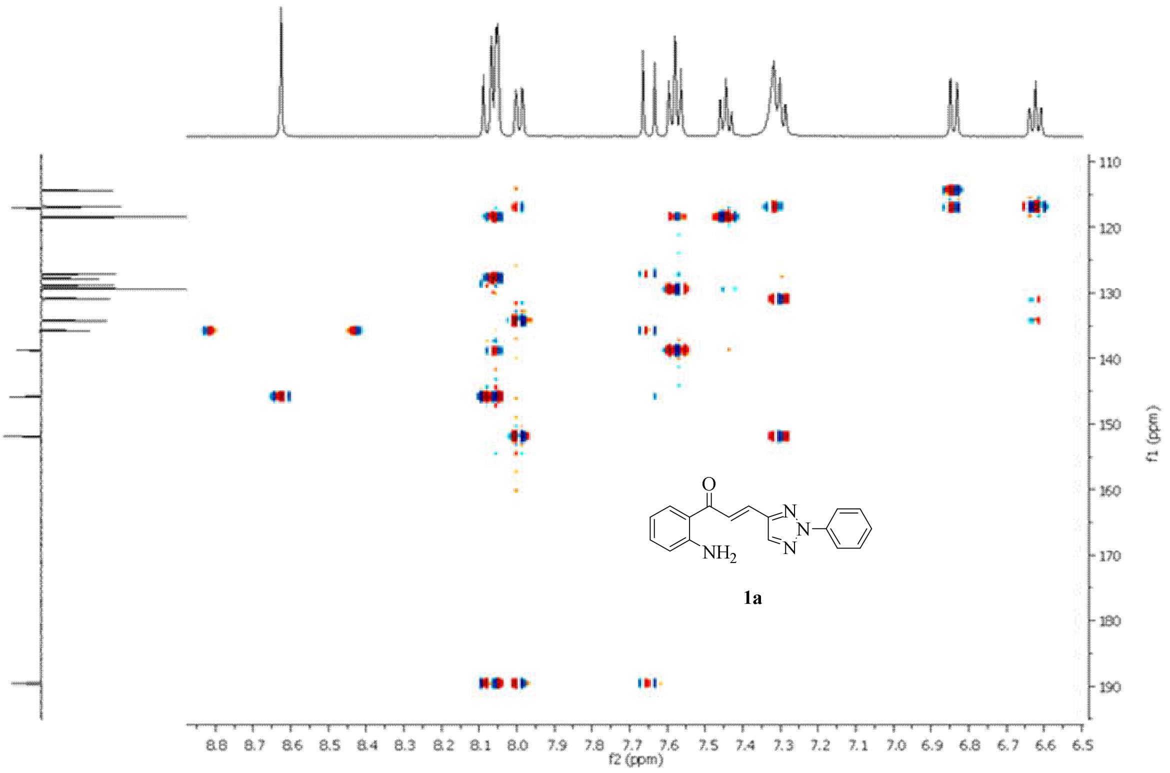

Figure S20. Expanded HSQC (DMSO-d6) of compound 1a

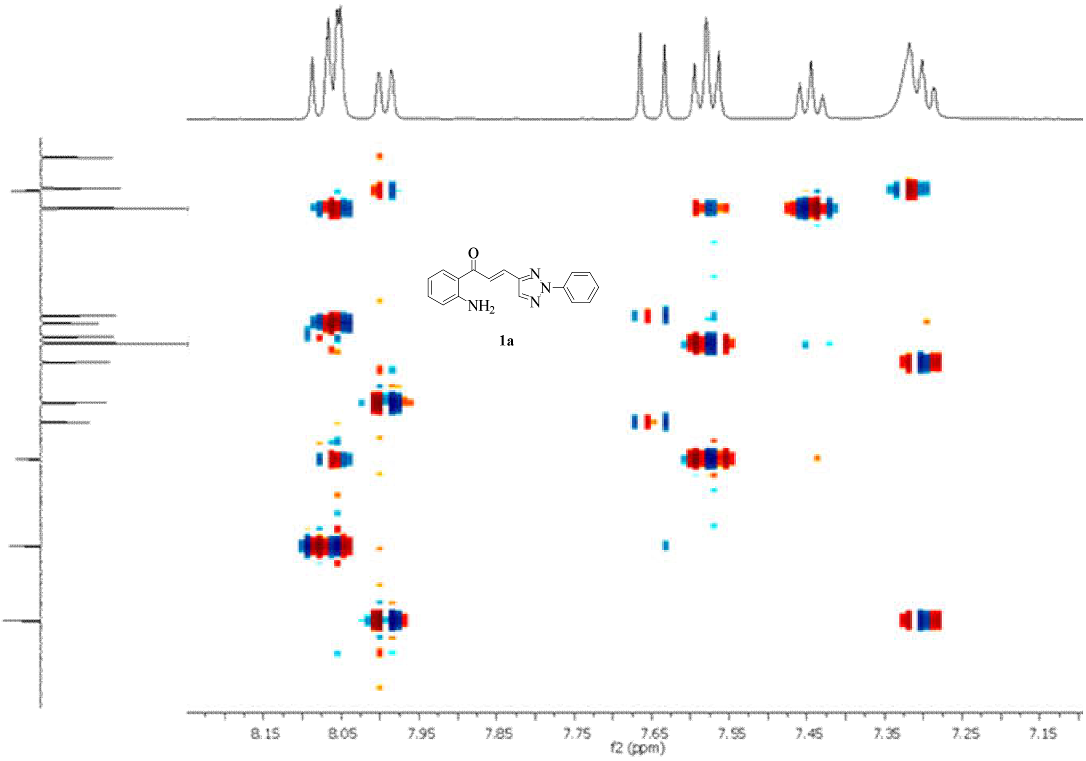

Figure S21. IR (KBr) of compound 1b

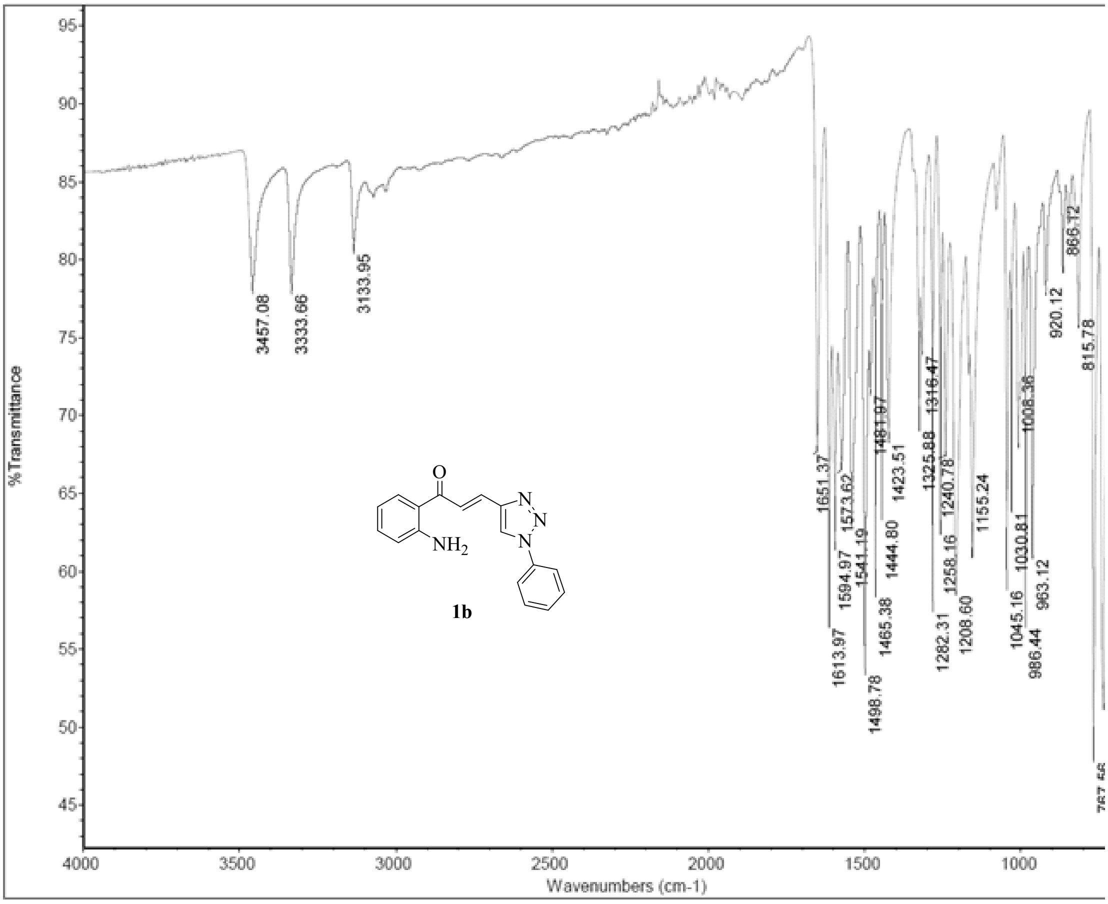

Figure S22. ^{1}H NMR (300 MHz. DMSO-d6) of compound 1b

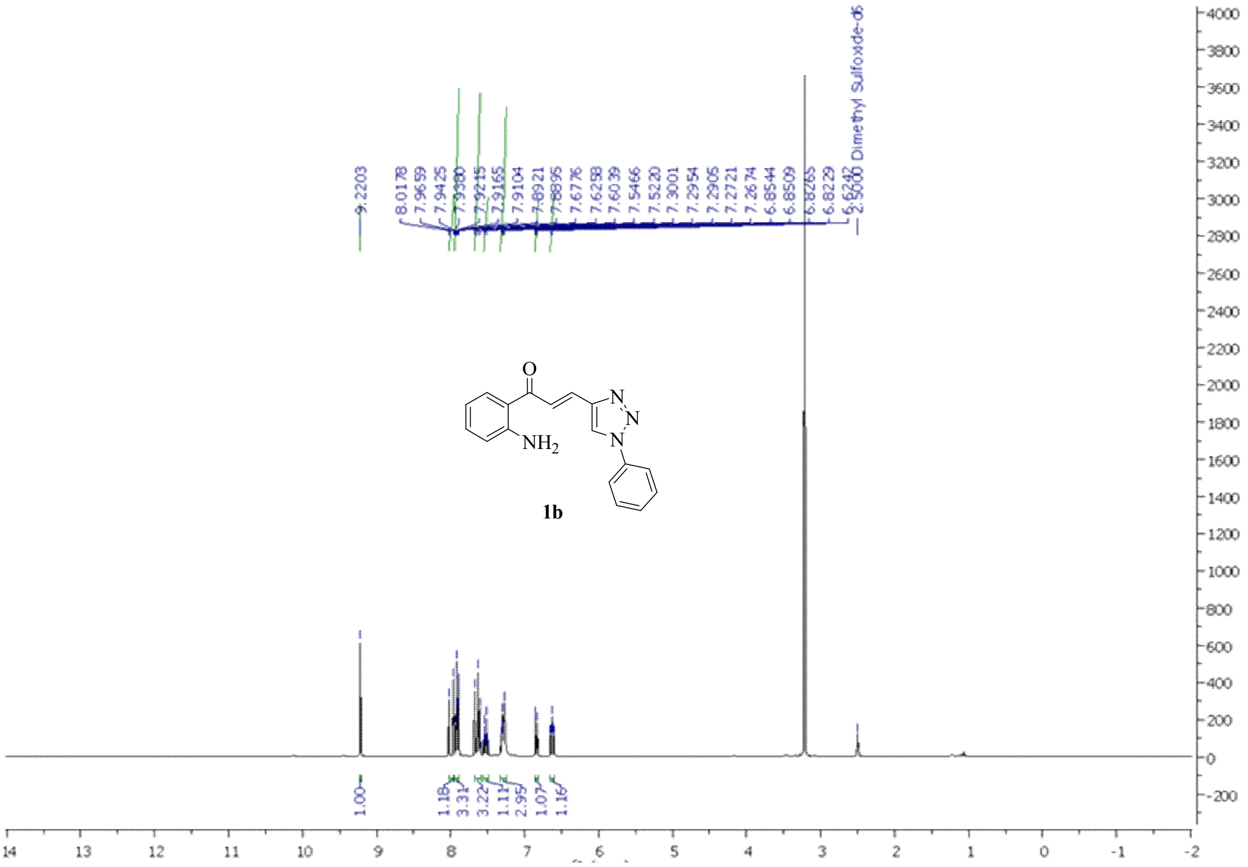

Figure S23. Expanded ^{1}H NMR (300 MHz. DMSO-d6) of compound 1b

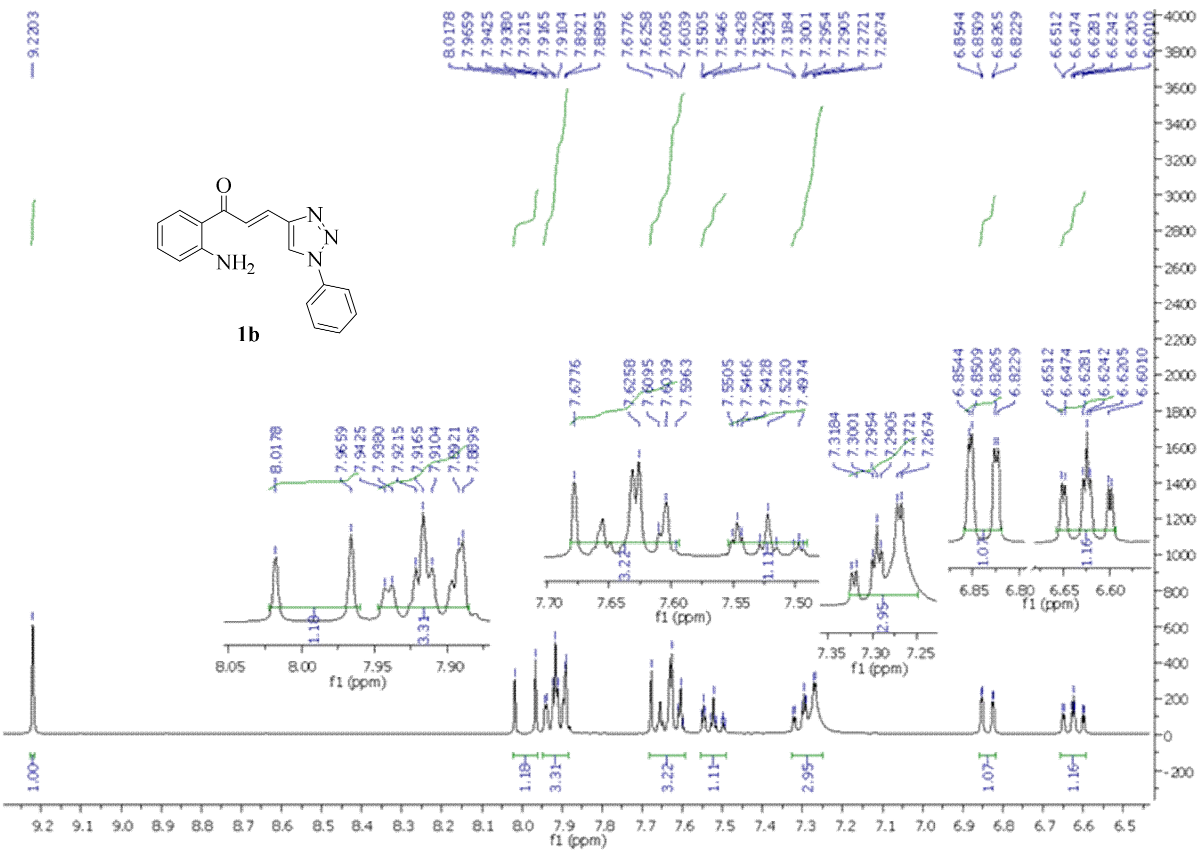

Figure S24. ^{13}C NMR/APT (75 MHz. DMSO-d6) of compound 1b

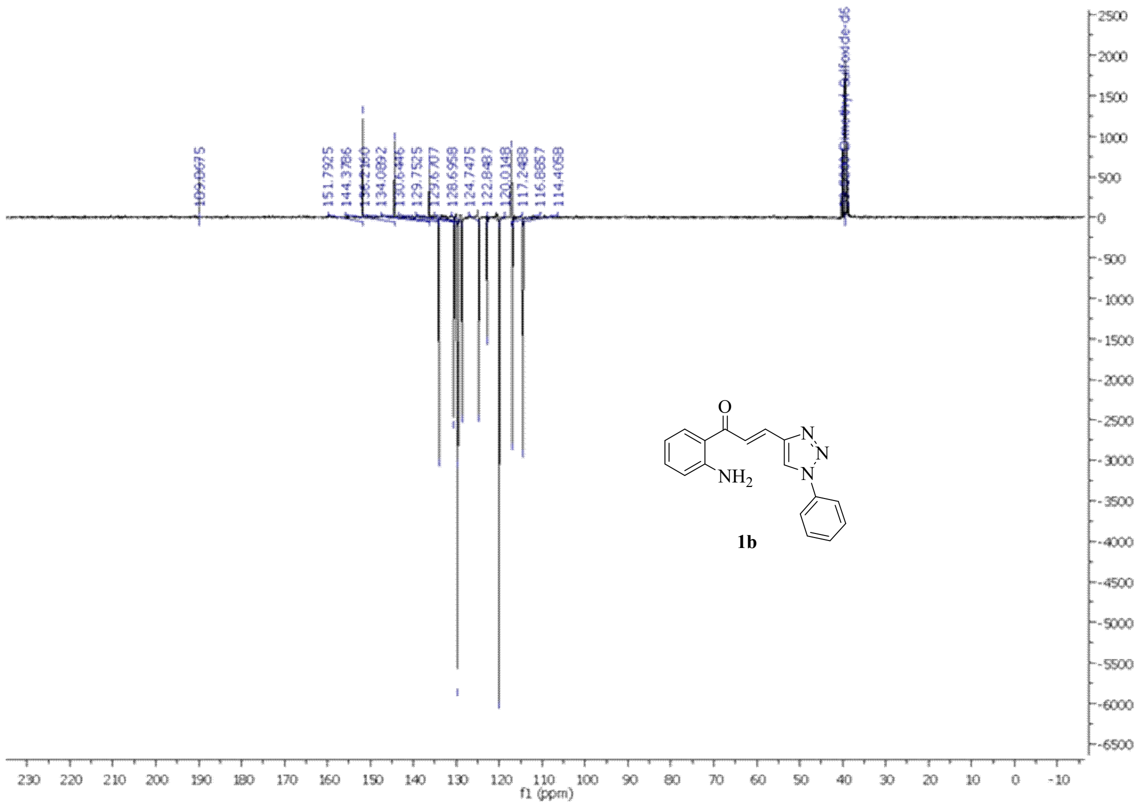

Figure S25. Expanded ^{13}C NMR/APT (75 MHz. DMSO-d6) of compound 1b

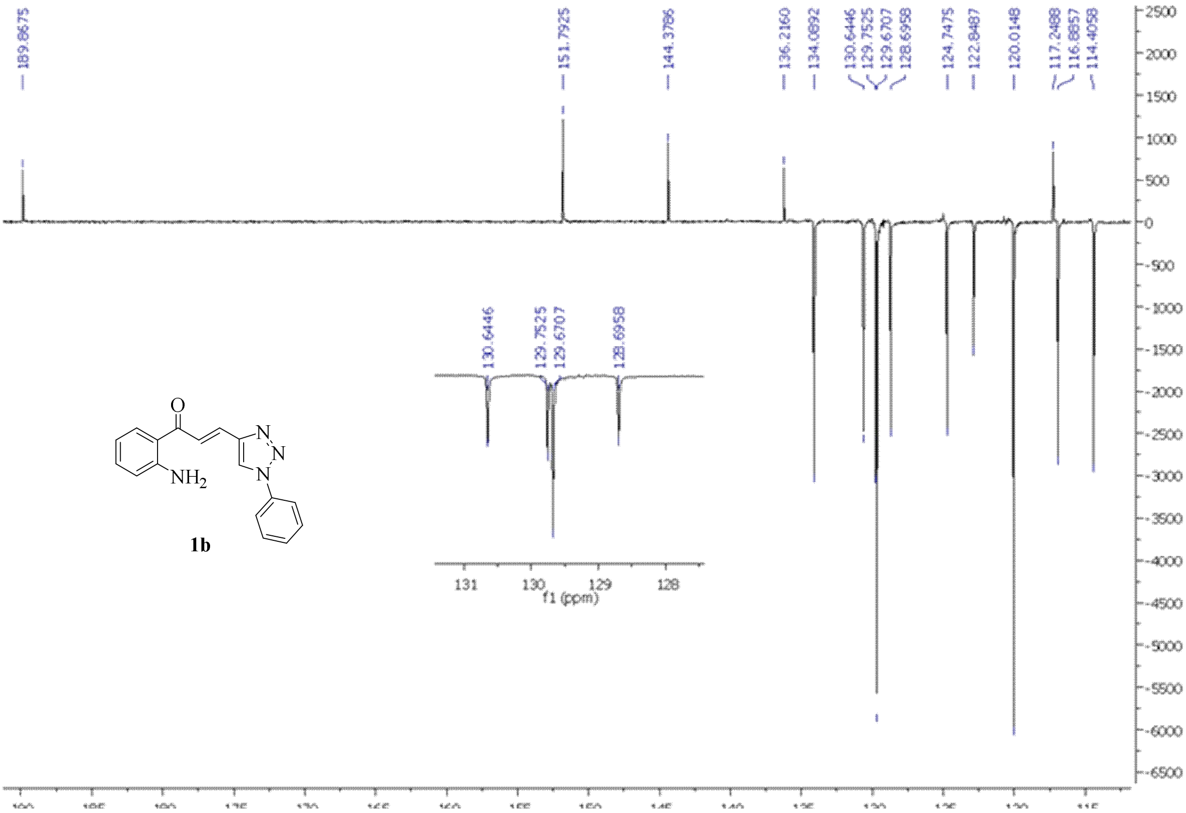

Figure S26. HSQC (DMSO-d6) of compound 1b

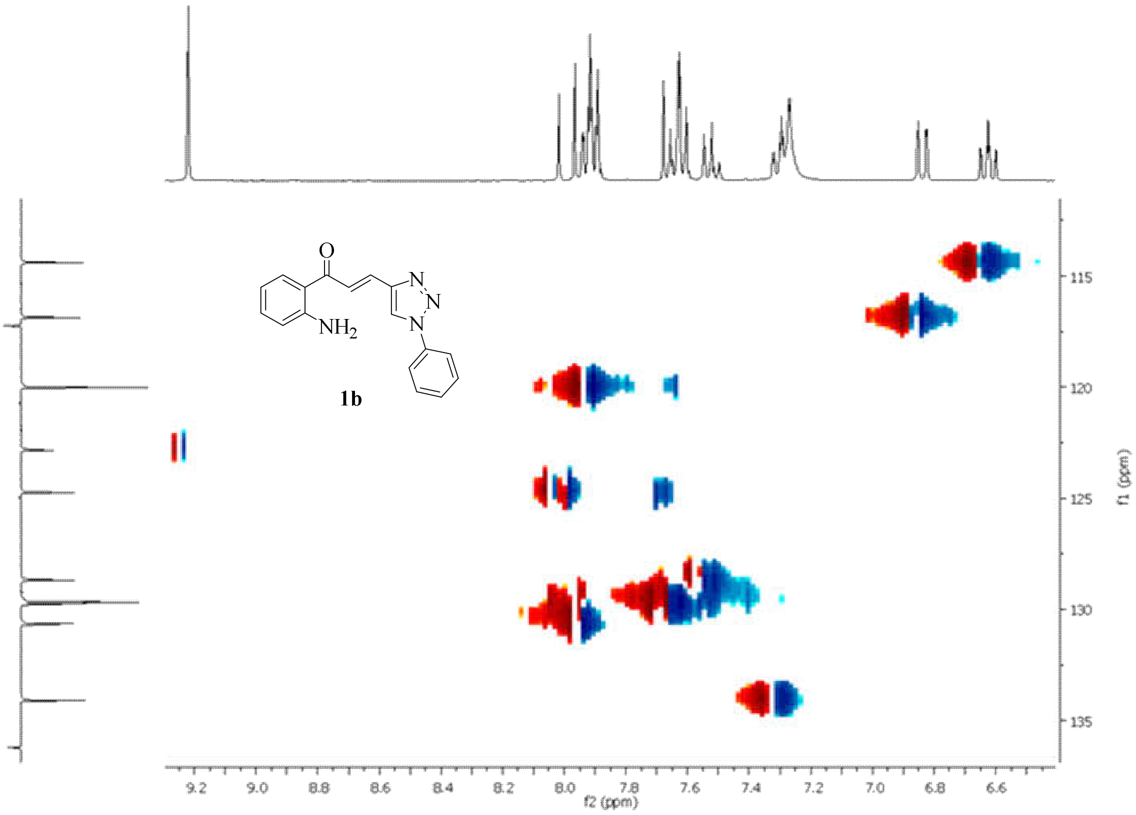

Figure S27. Expanded HSQC (DMSO-d6) of compound 1b

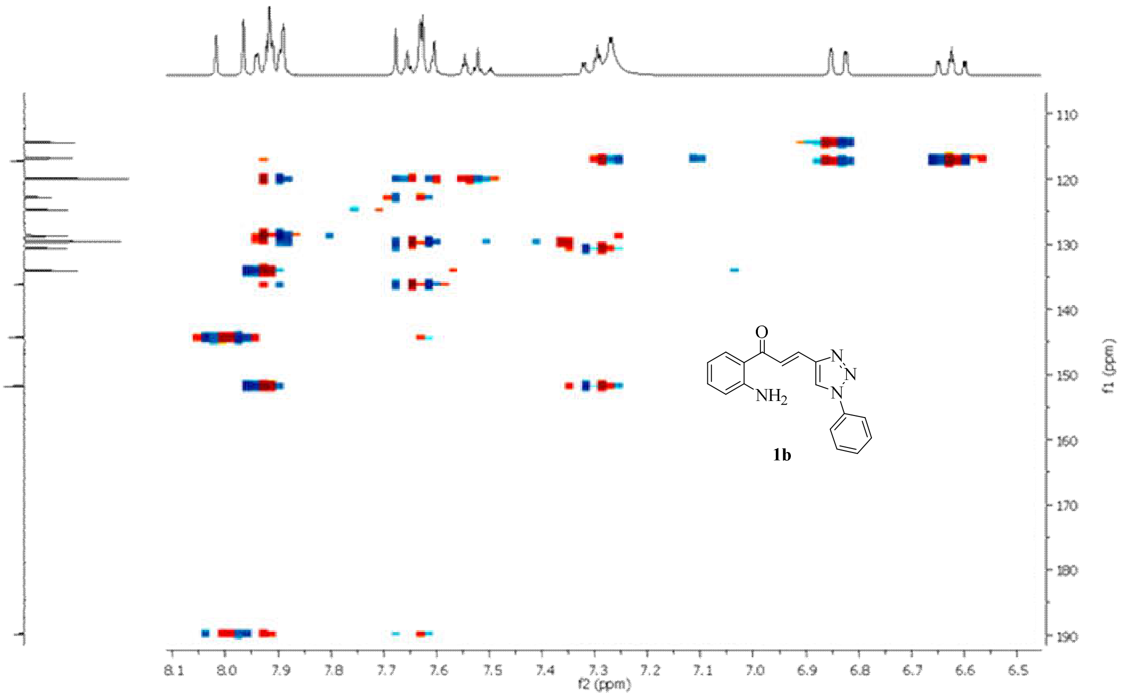

Figure S28. IR (KBr) of compound 1c

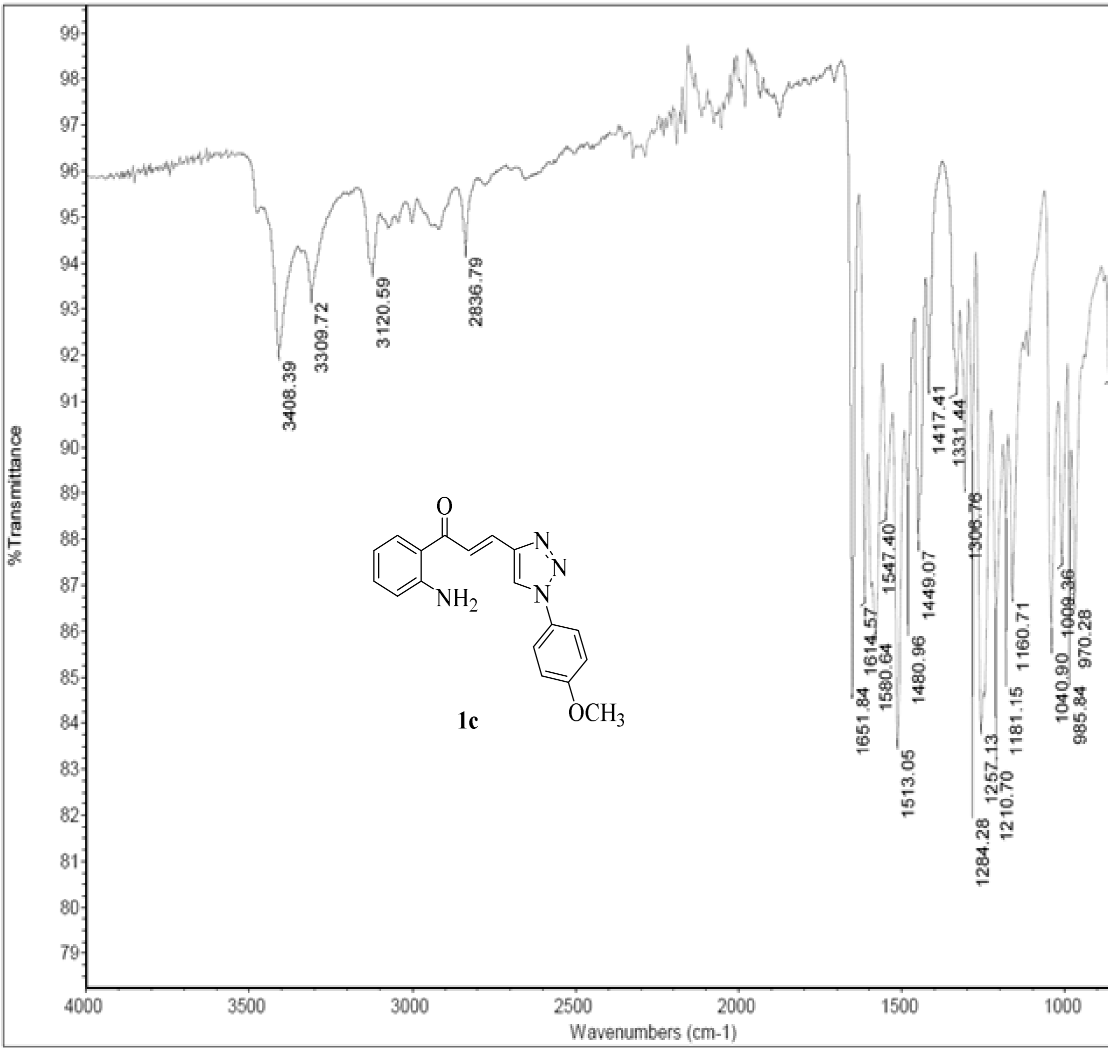

Figure S29. ^{1}H NMR (300 MHz. DMSO-d6) of compound 1c

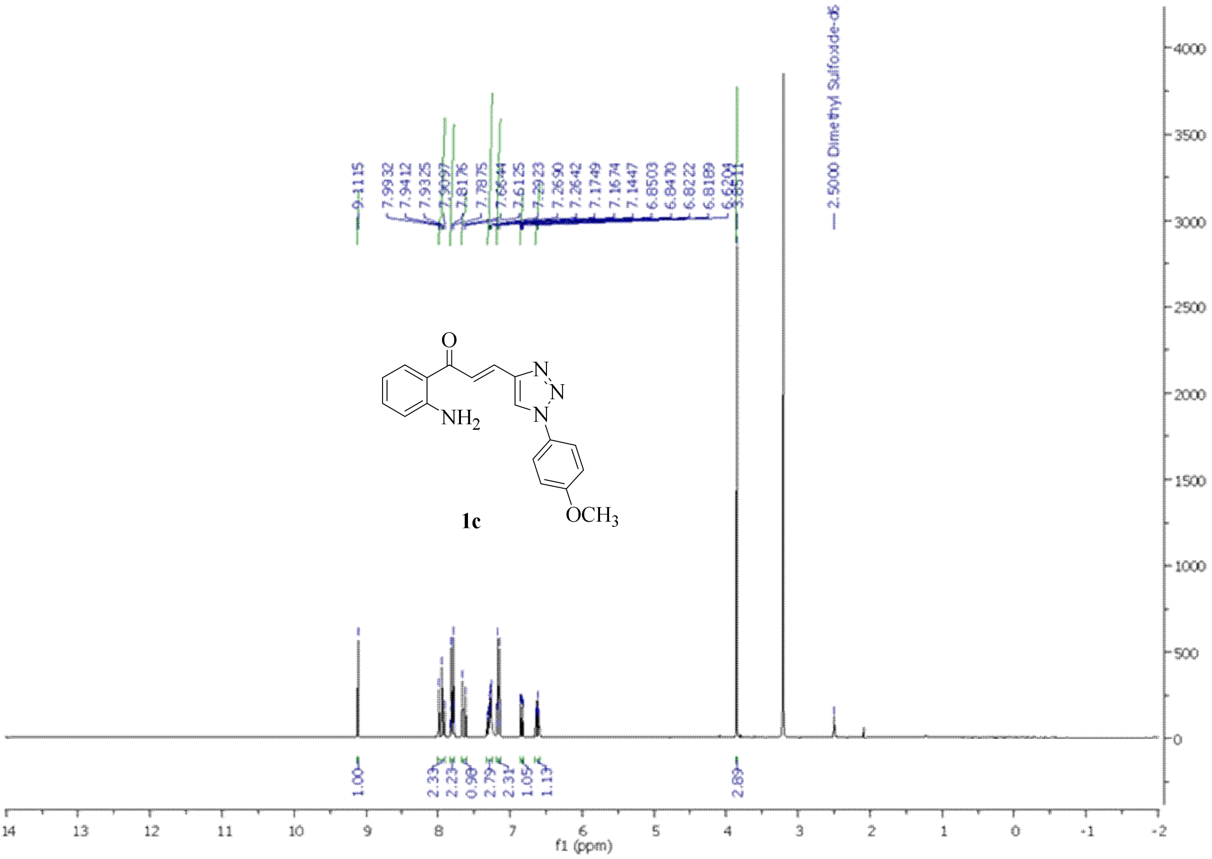

Figure S30. Expanded ^{1}H NMR (300 MHz. DMSO-d6) of compound 1c

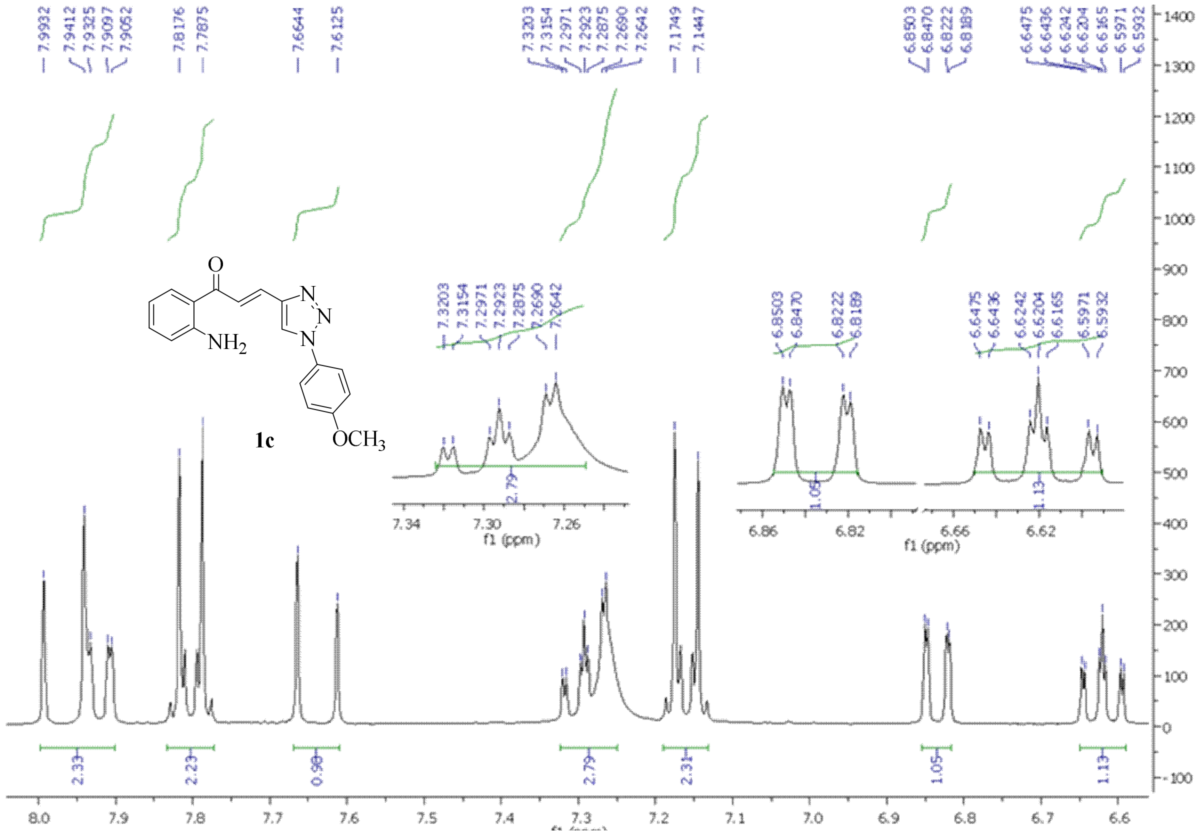

Figure S31. ^{13}C NMR/APT (75 MHz. DMSO-d6) of compound 1c

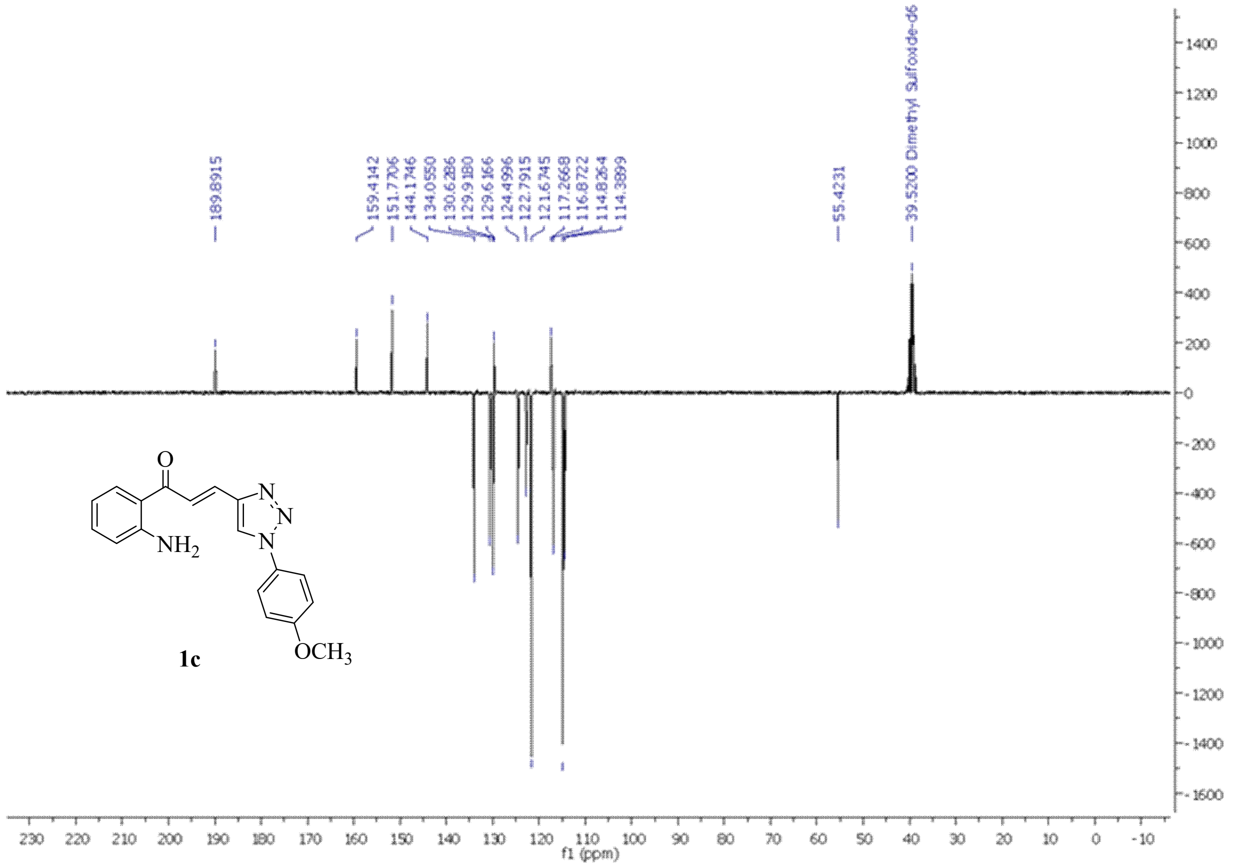

Figure S32. Expanded ^{13}C NMR/APT (75 MHz. DMSO-d6) of compound 1c

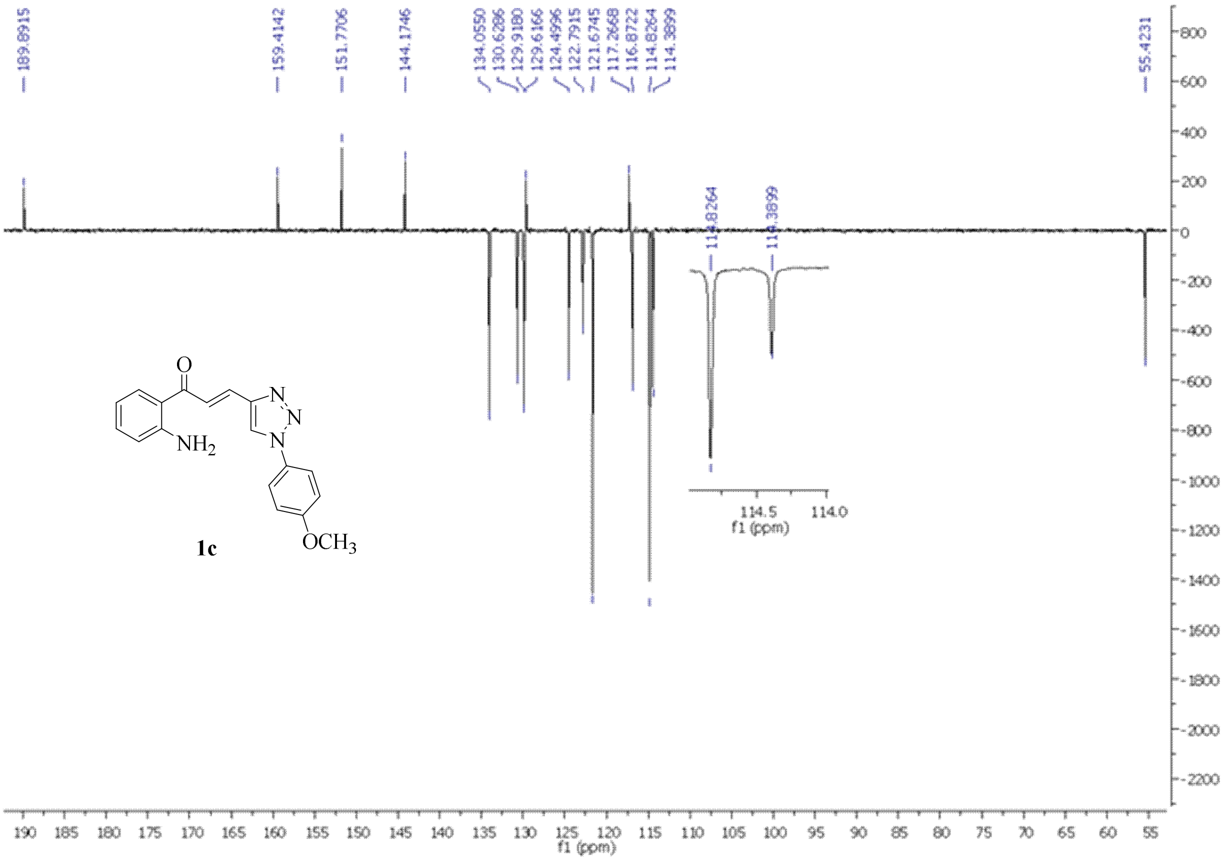

Figure S33. IR (KBr) of compound 1d

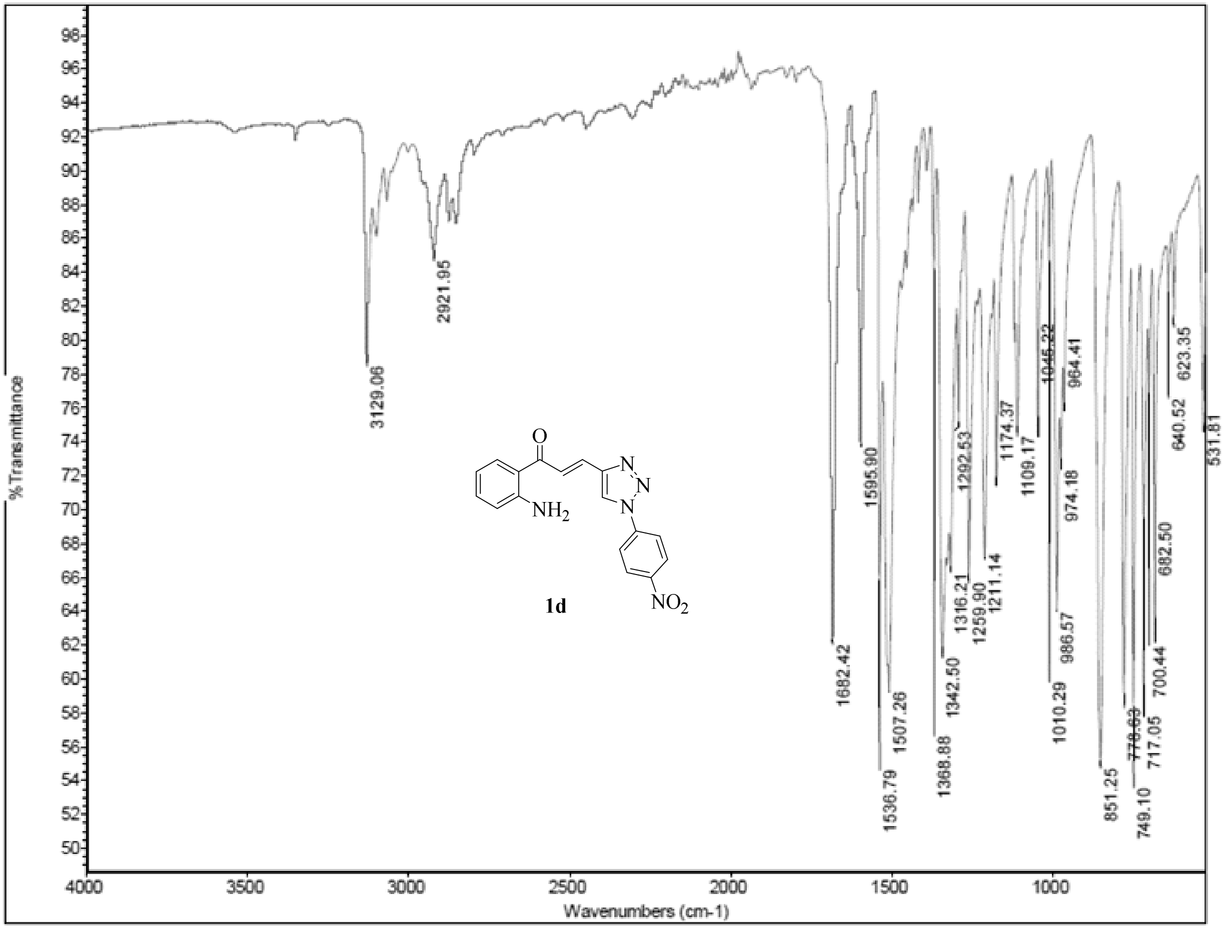

Figure S34. ^{1}H NMR (500 MHz. DMSO-d6) of compound 1d

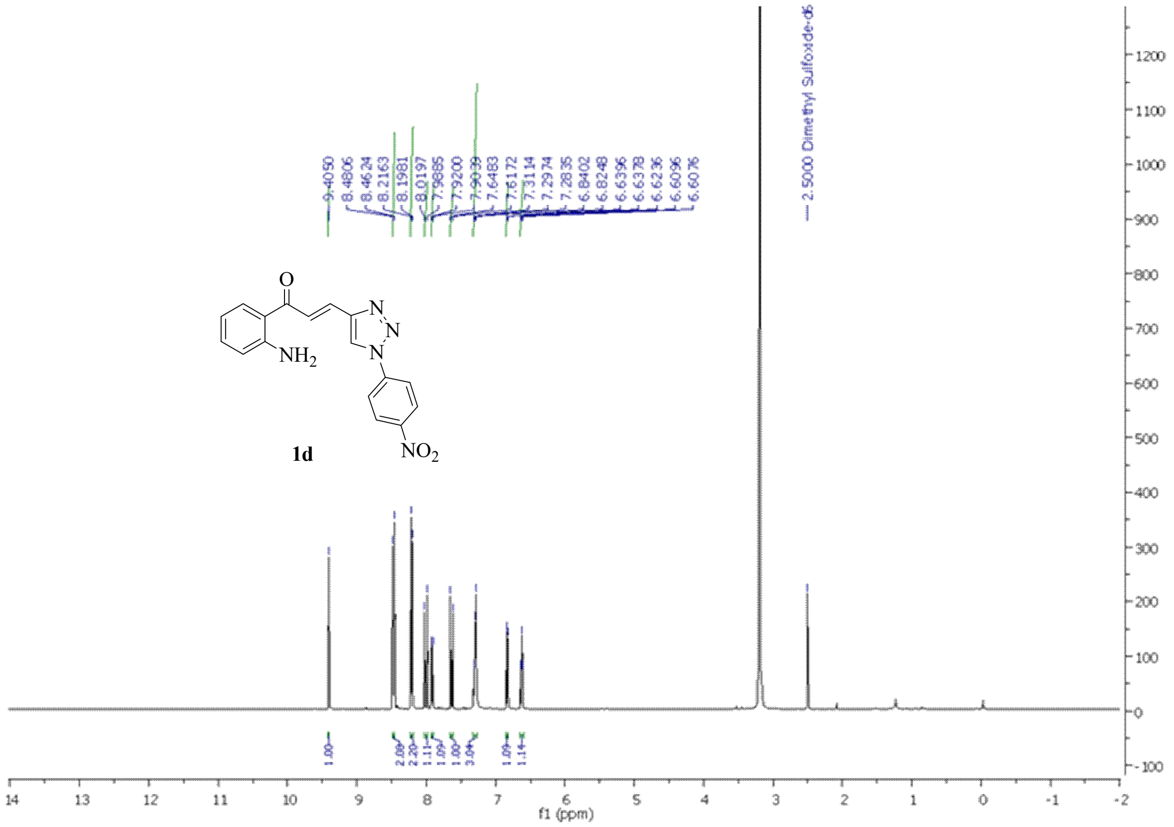

Figure S35. Expanded ^{1}H NMR (500 MHz. DMSO-d6) of compound 1d

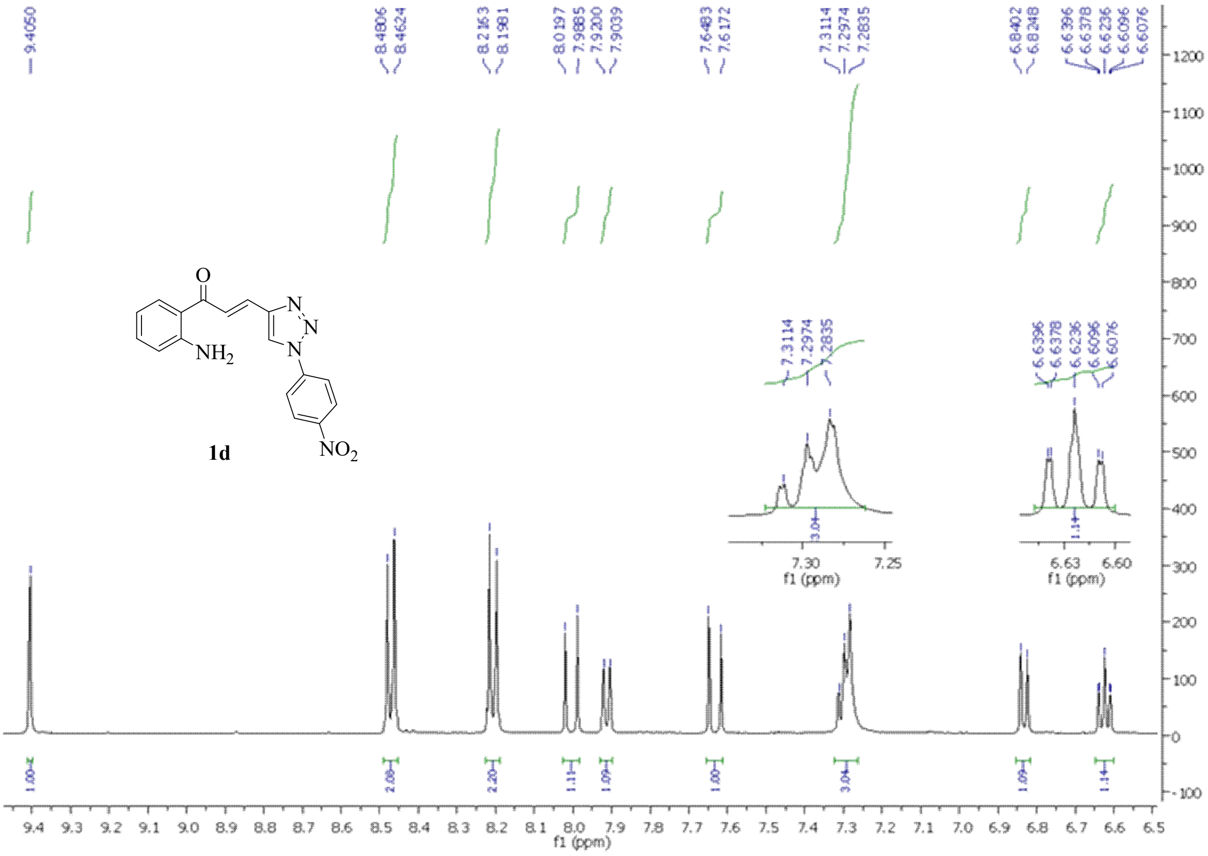

Figure S36. ^{13}C NMR/APT (125 MHz. DMSO-d6) of compound 1d

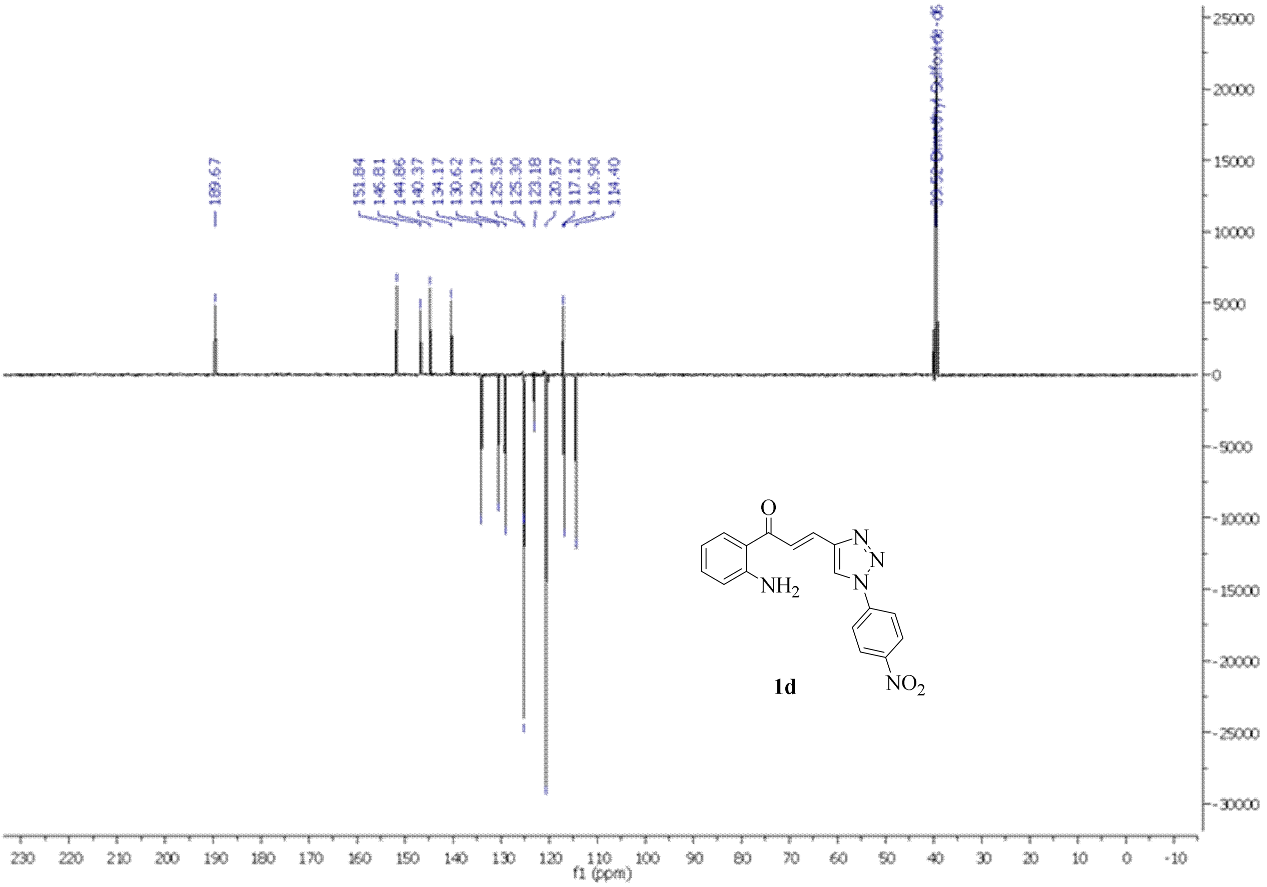

Figure S37. Expanded ^{13}C NMR/APT (75 MHz. DMSO-d6) of compound 1d

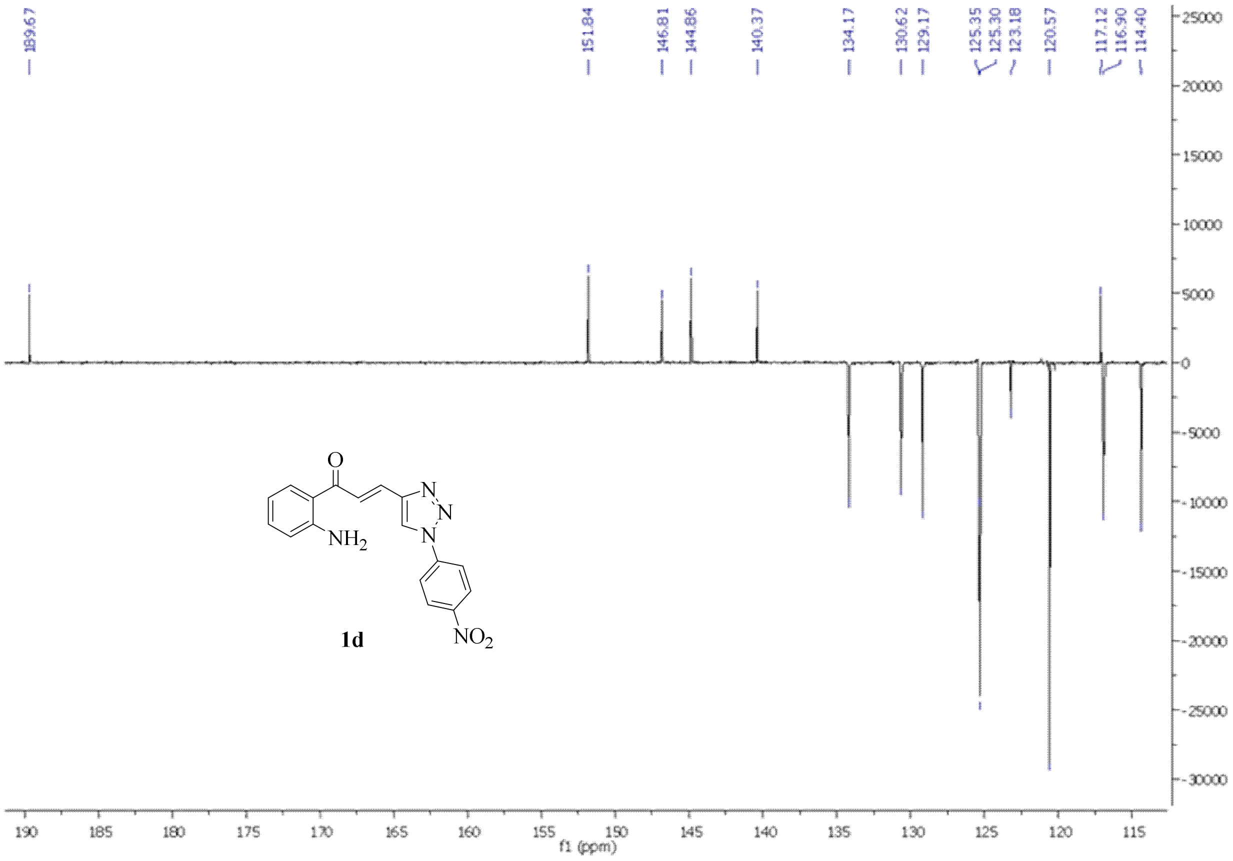

Figure S38. IR (KBr) of compound 1e

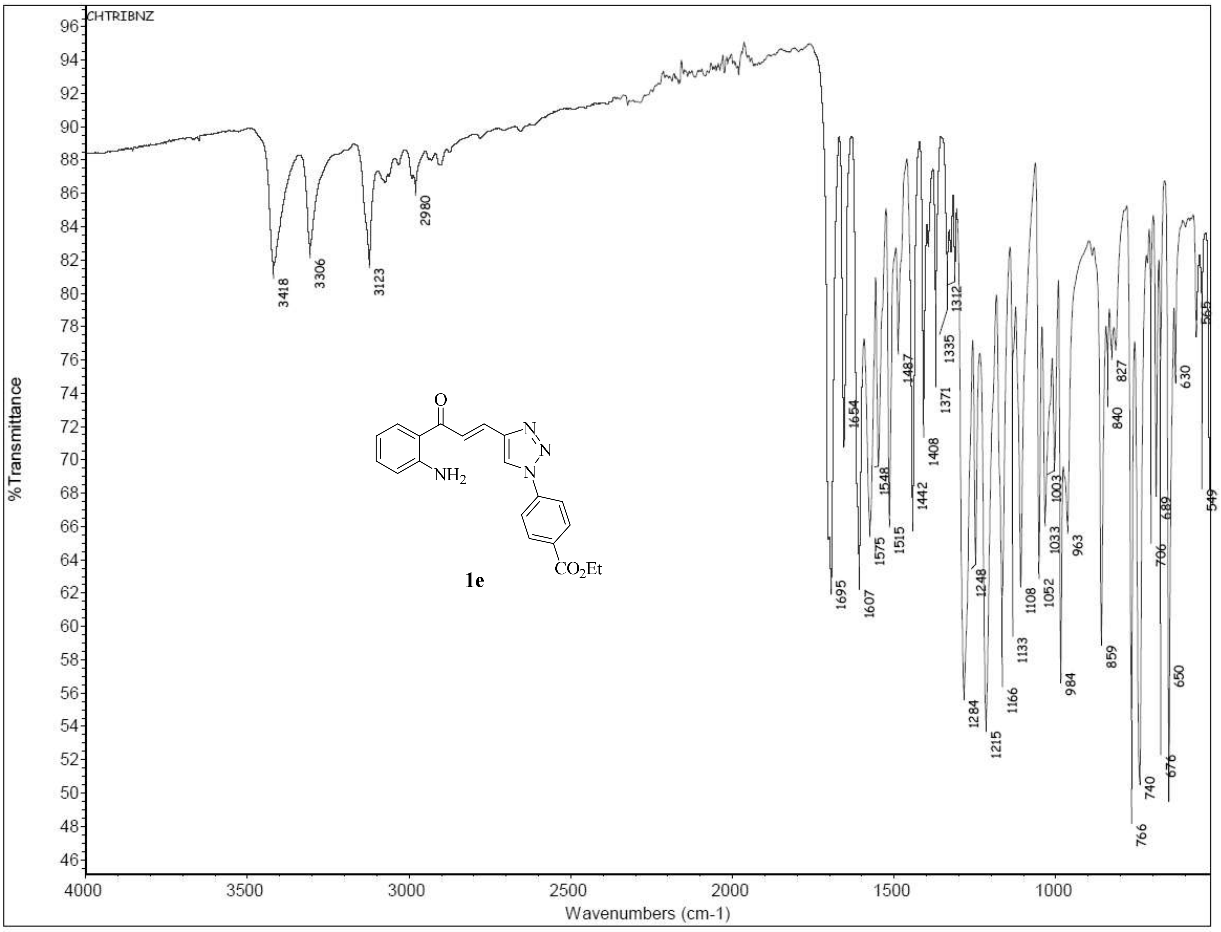

Figure S39. ^{1}H NMR (500 MHz. DMSO-d6) of compound 1e

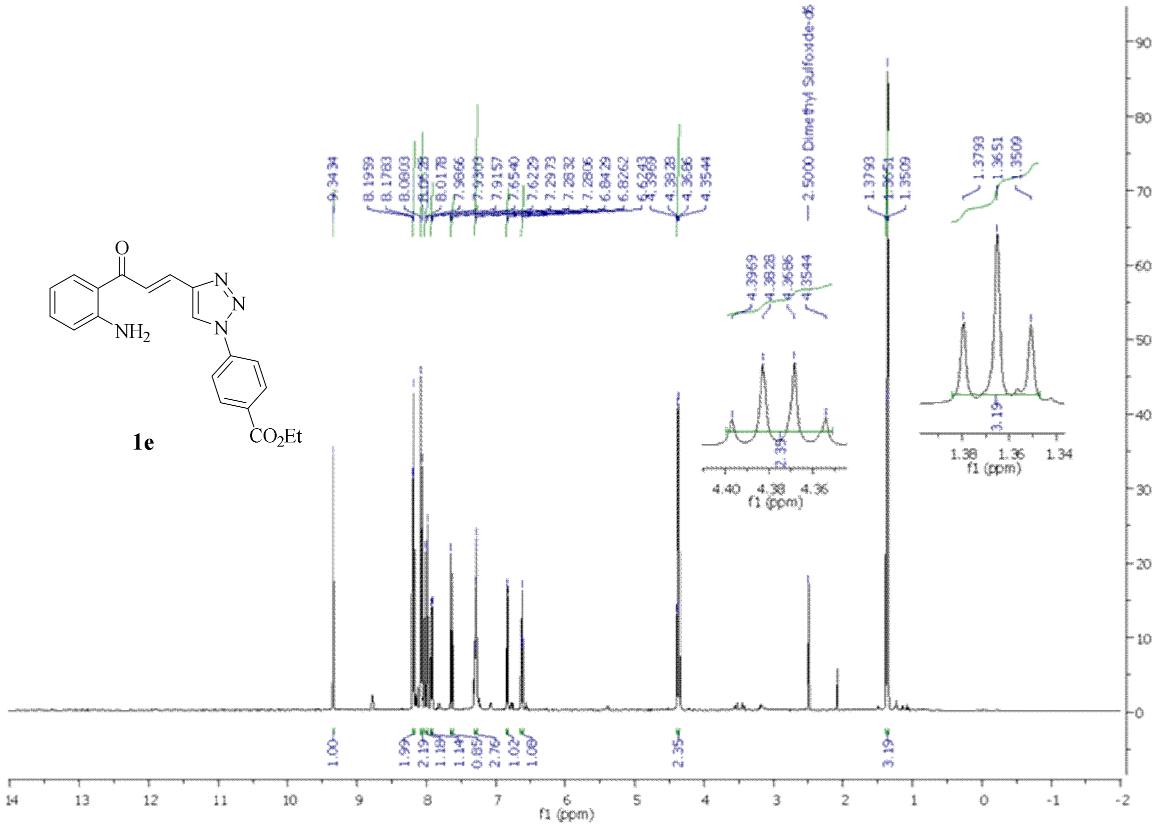

Figure S40. Expanded ^{1}H NMR (500 MHz. DMSO-d6) of compound 1e

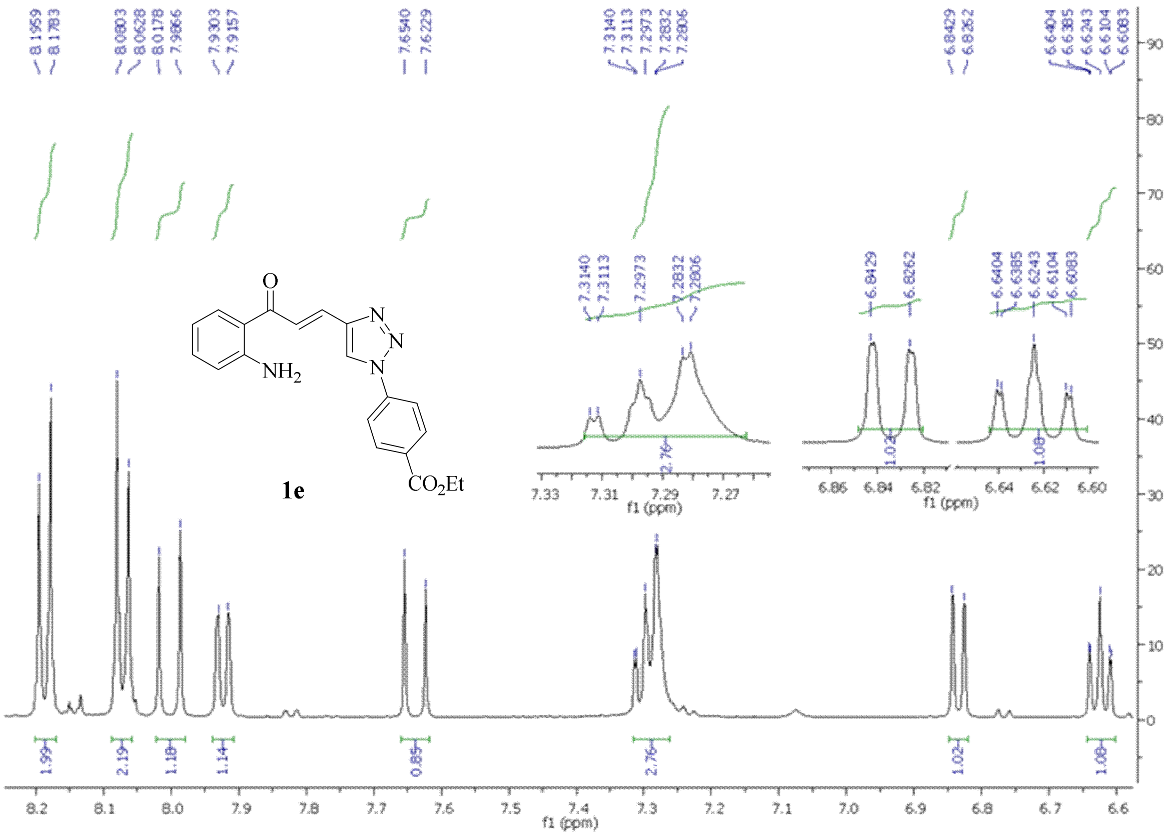

Figure S41. ^{13}C NMR/APT (125 MHz. DMSO-d6) of compound 1e

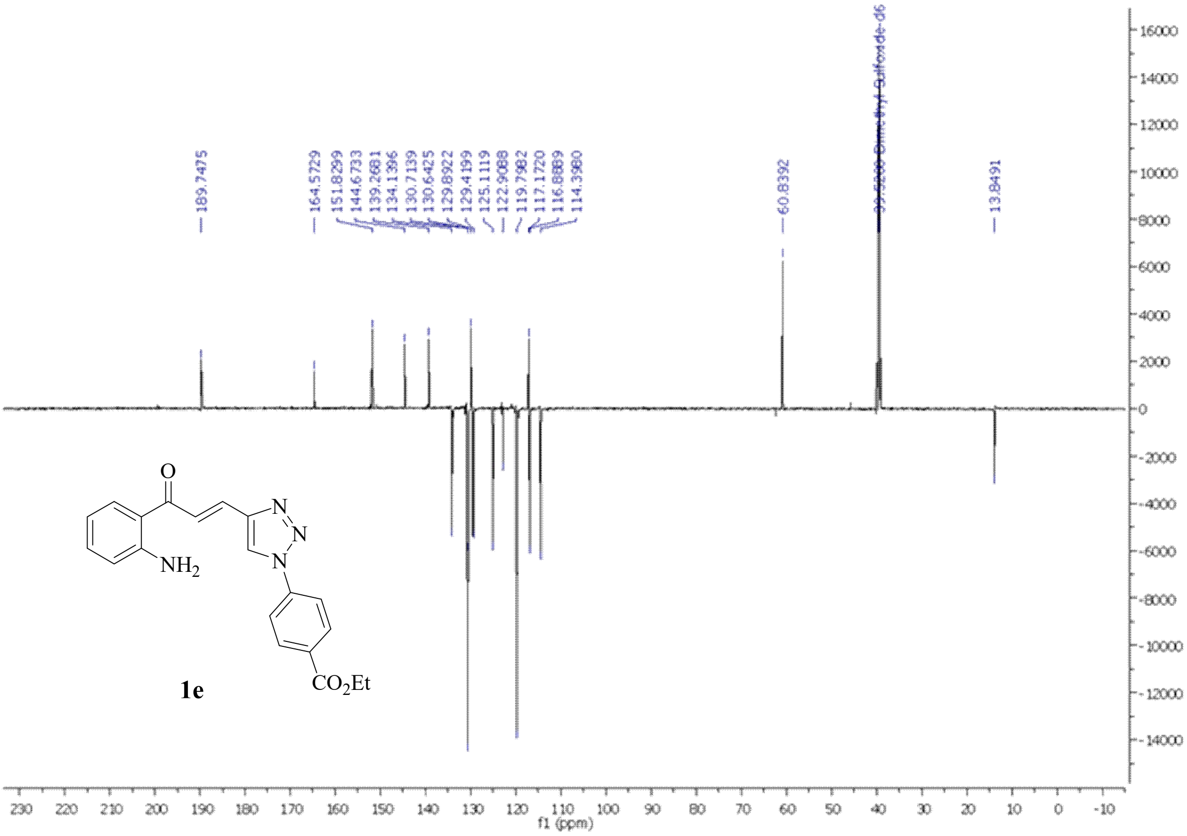

Figure S42. Expanded ^{13}C NMR/APT (125 MHz. DMSO-d6) of compound 1e

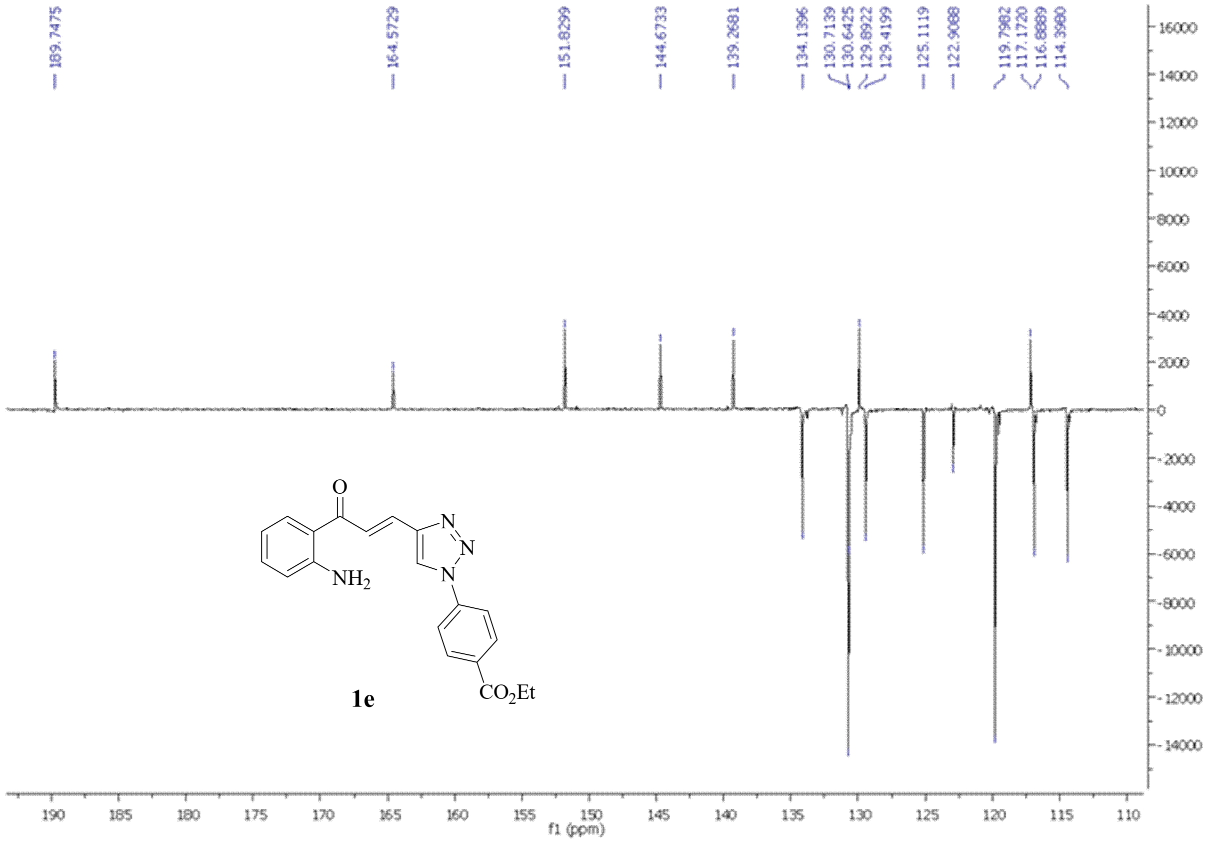

Figure S43. IR (KBr) of compound 1f

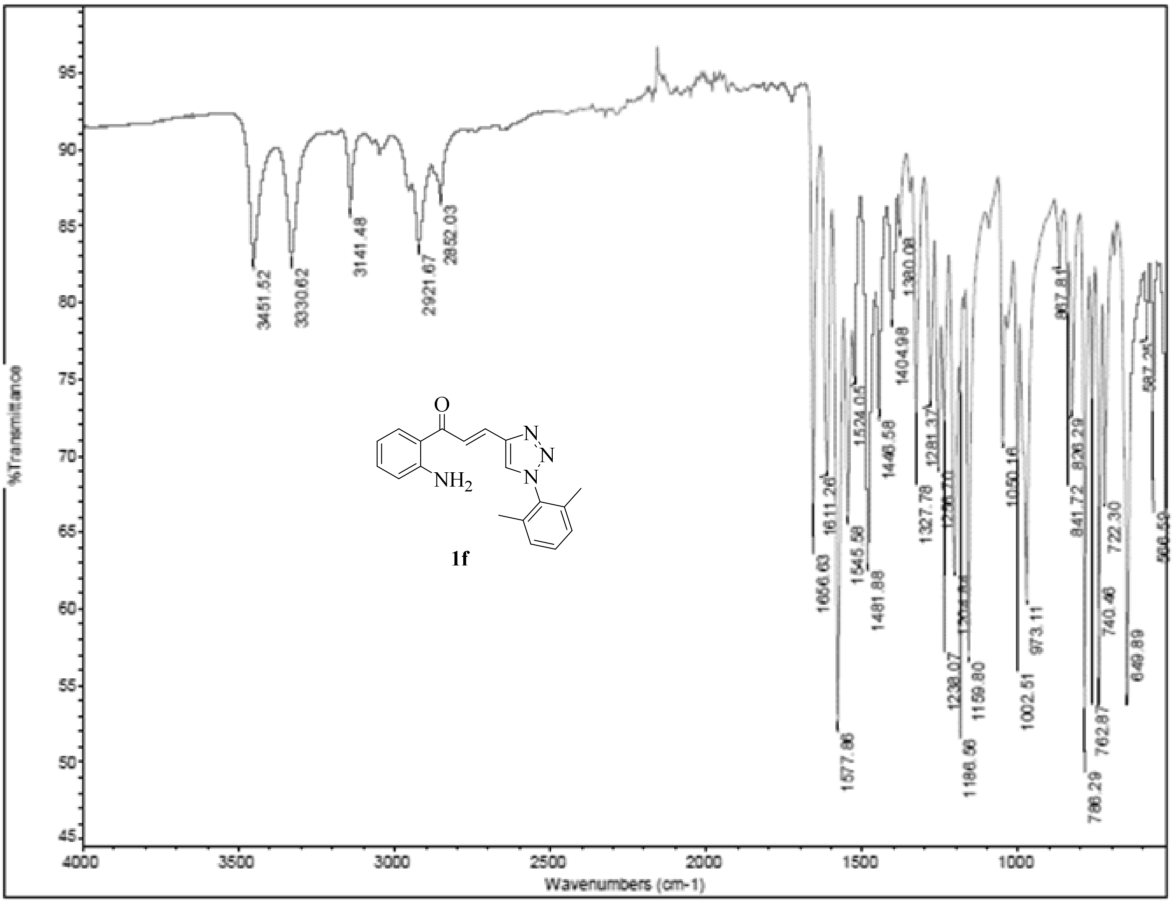

Figure S44. ^{1}H NMR (500 MHz. DMSO-d6)) of compound 1f

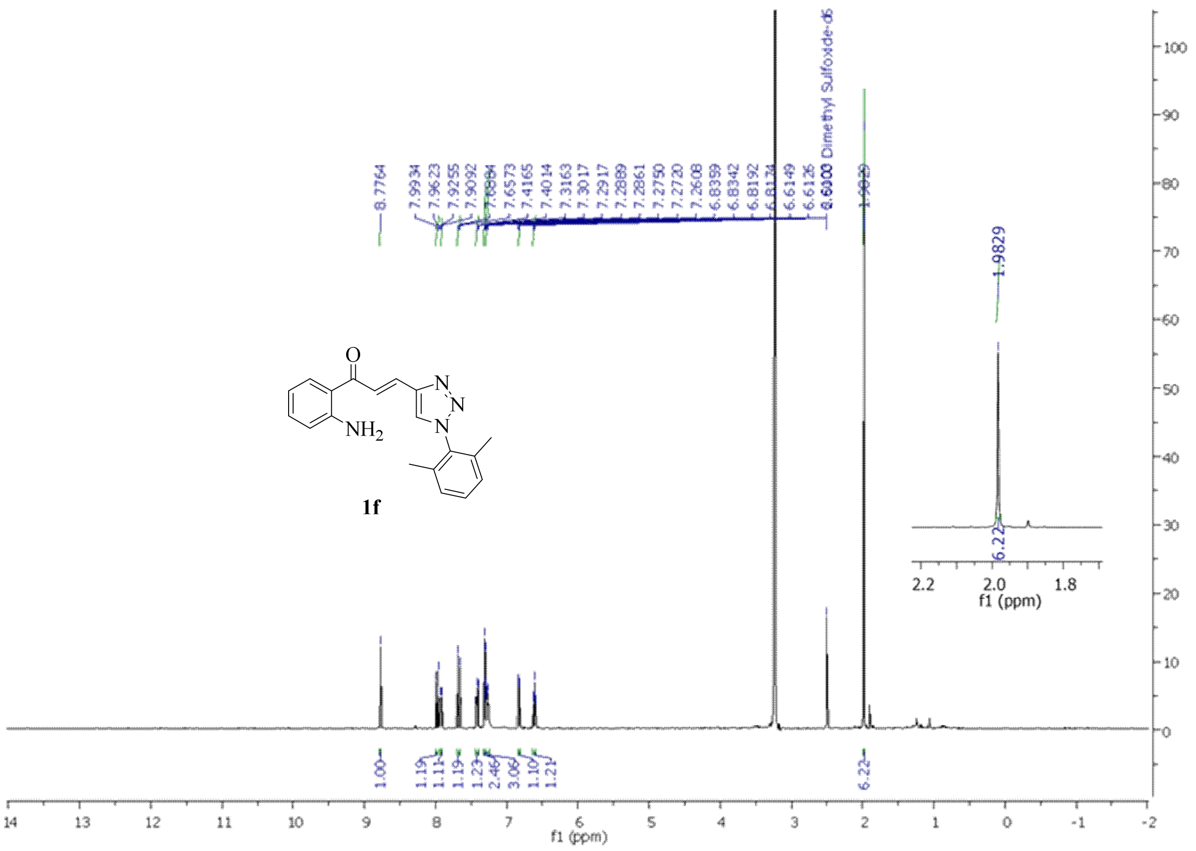

Figure S45. Expanded ^{1}H NMR (500 MHz. DMSO-d6)) of compound 1f

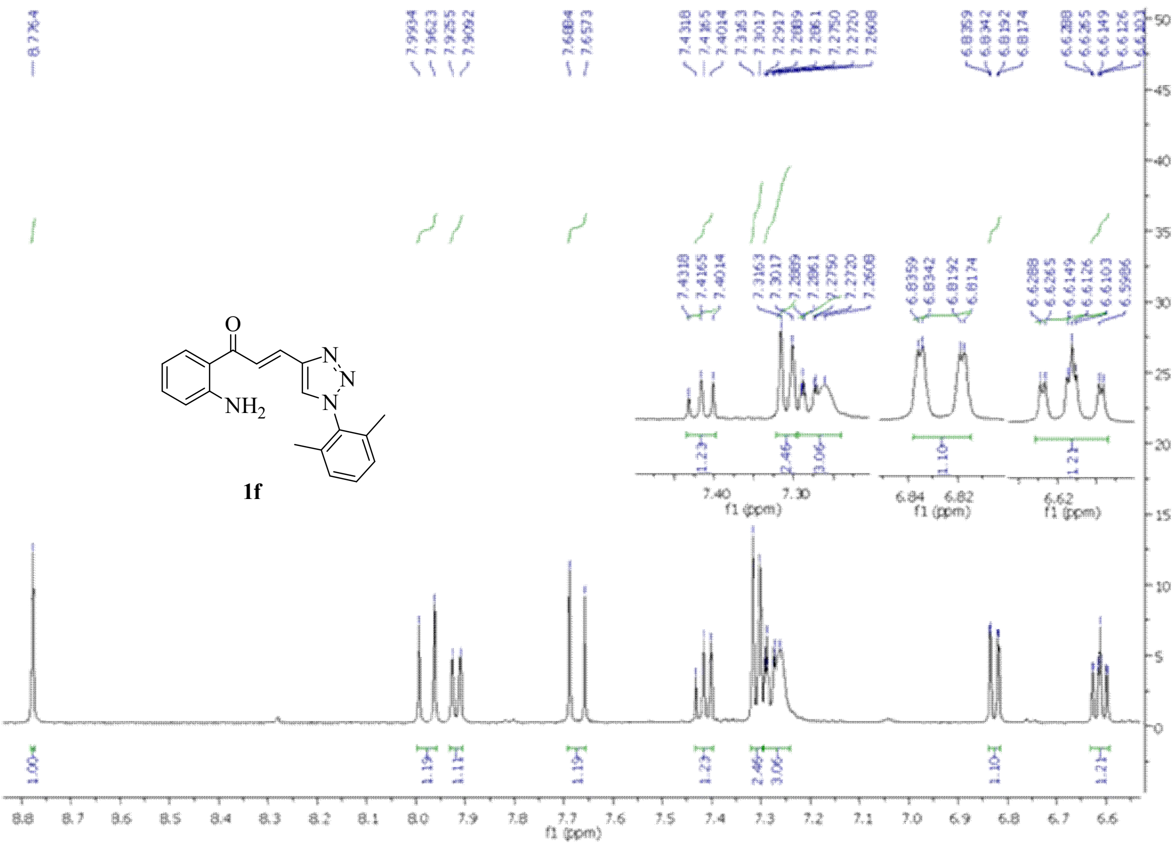

Figure S46. COSY (DMSO-d6) of compound 1f

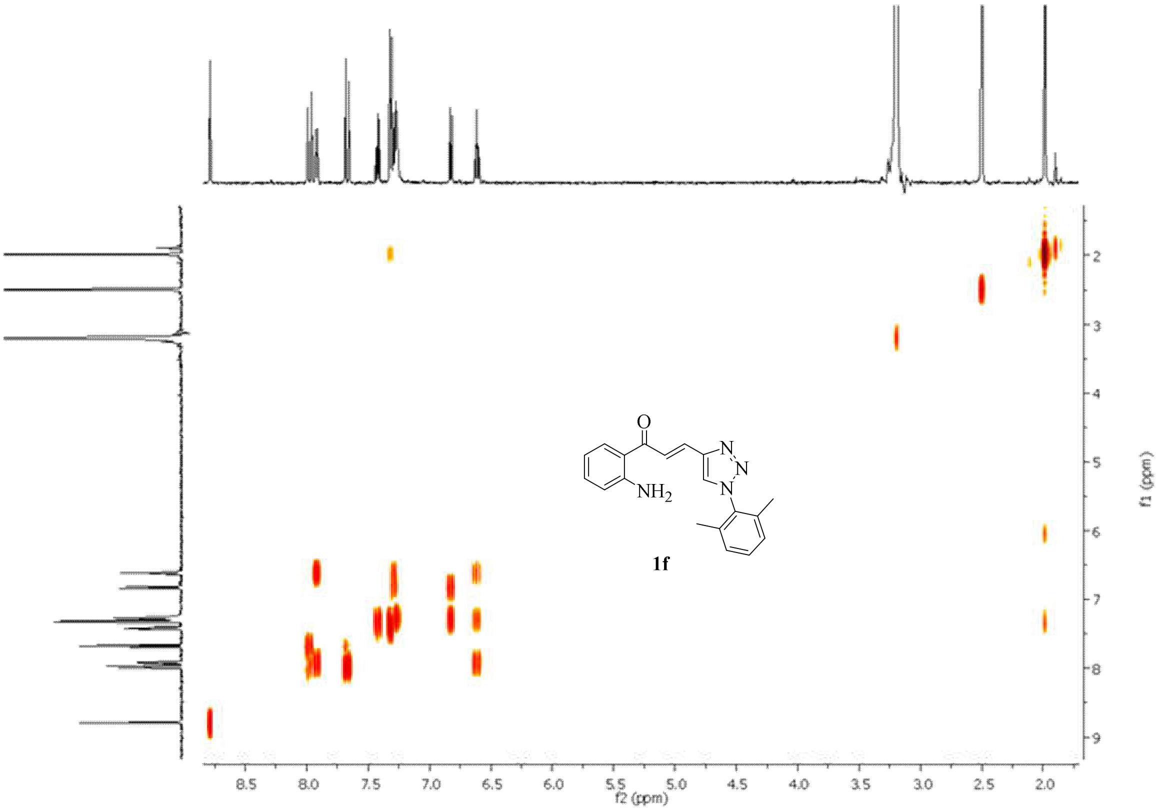

Figure S47. ^{13}C NMR/APT (125 MHz. DMSO-d6) of compound 1f

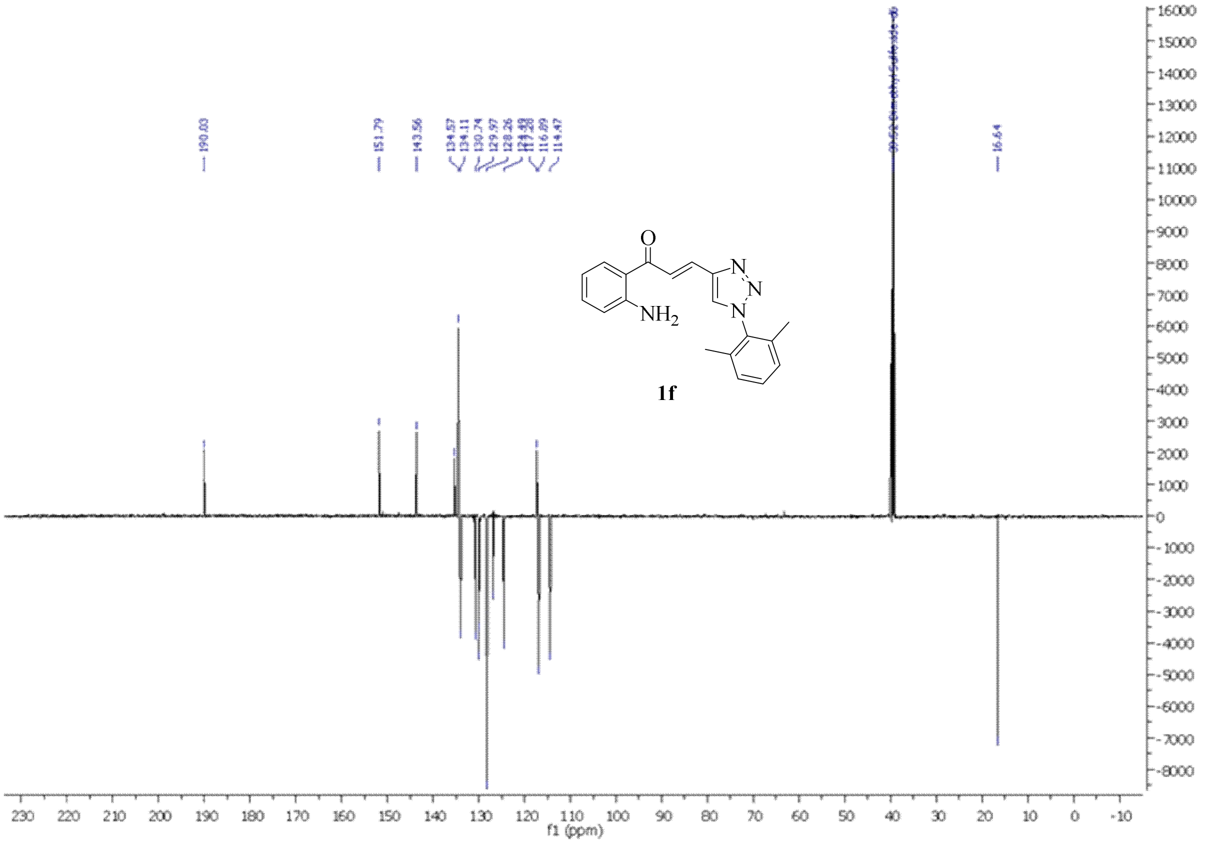

Figure S48. Expanded ^{13}C NMR/APT (125 MHz. DMSO-d6) of compound 1f

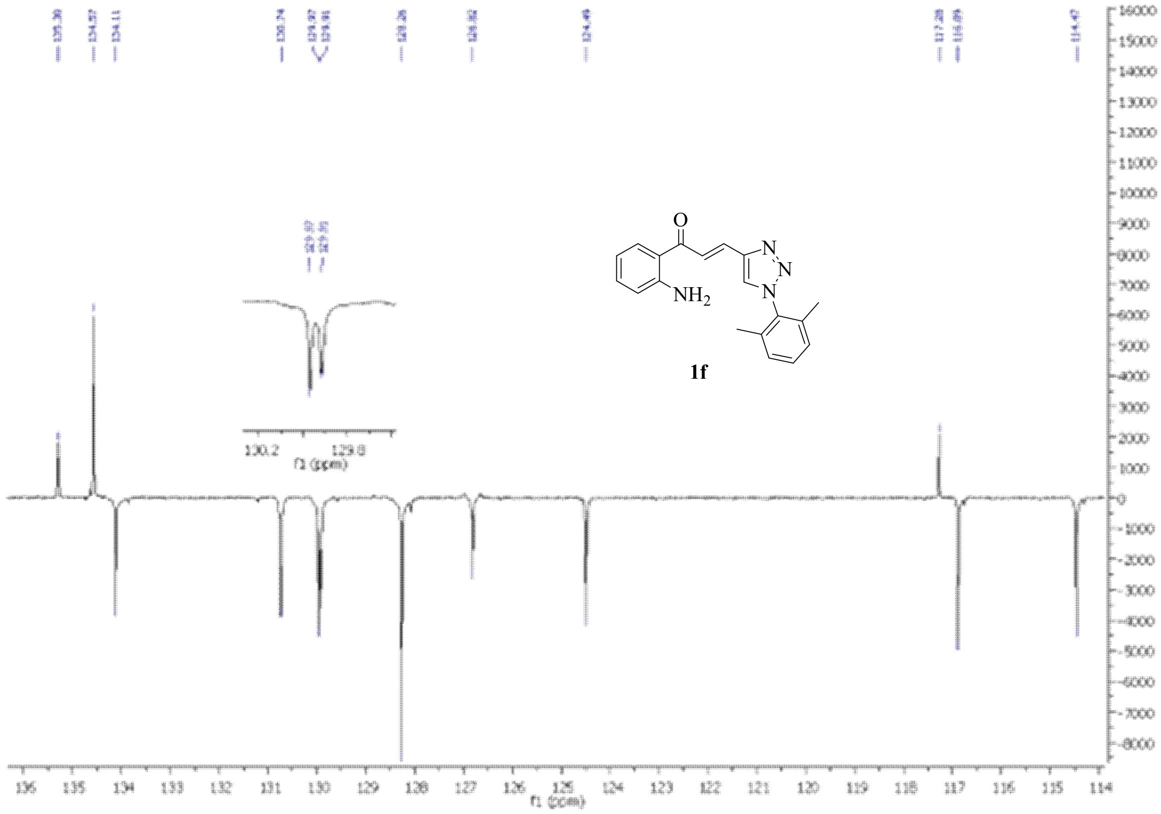

Figure S49. HSQC (DMSO-d6) of compound 1f

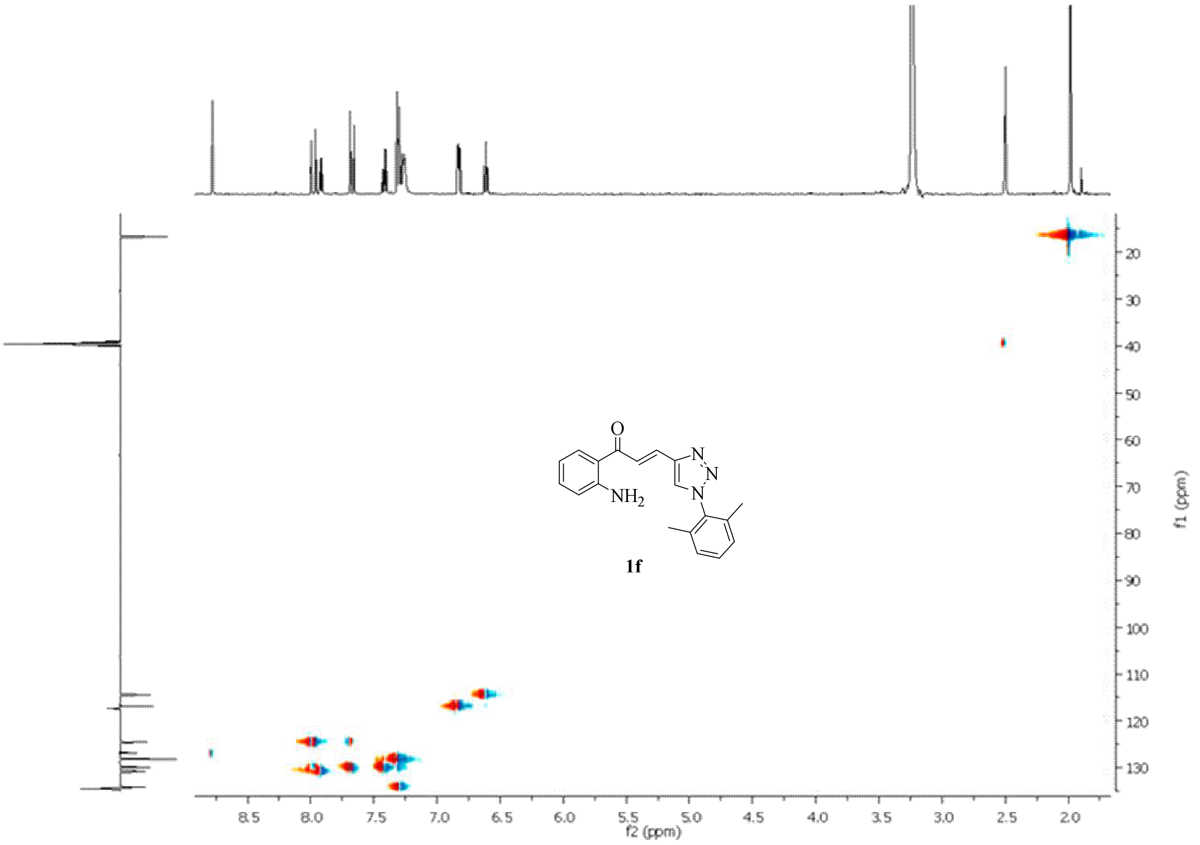

Figure S50. Expanded HSQC (DMSO-d6) of compound 1f

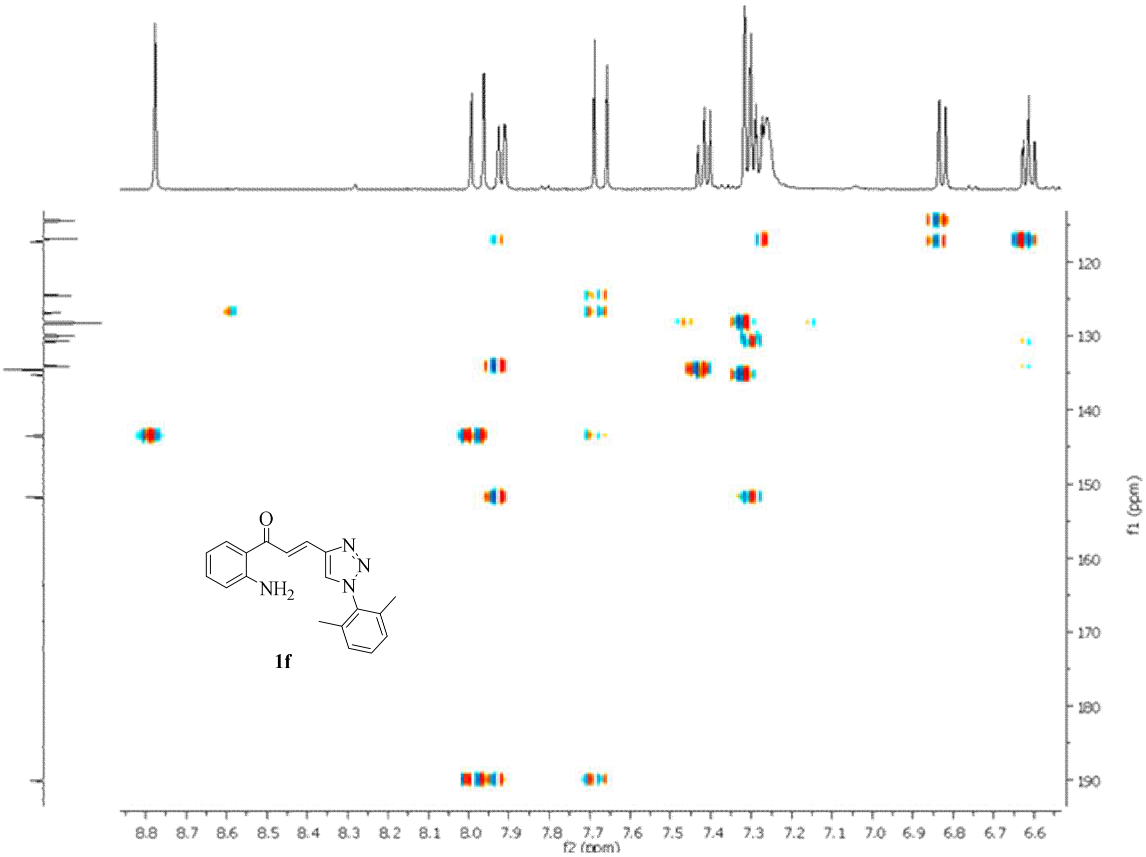

Supplement: Supplementary file 1 [file biomedicines-11-01711-s001.zip › biomedicines-2444097-supplementary.pdf]
